# Bovine Nebovirus Interacts with a Wide Spectrum of Histo-Blood Group Antigens

**DOI:** 10.1128/JVI.02160-17

**Published:** 2018-04-13

**Authors:** Eun-Hyo Cho, Mahmoud Soliman, Mia Madel Alfajaro, Ji-Yun Kim, Ja-Young Seo, Jun-Gyu Park, Deok-Song Kim, Yeong-Bin Baek, Mun-Il Kang, Sang-Ik Park, Jacques Le Pendu, Kyoung-Oh Cho

**Affiliations:** a Laboratory of Veterinary Pathology, College of Veterinary Medicine, Chonnam National University, Gwangju, Republic of Korea; b CRCINA, Inserm, Université d'Angers, Université de Nantes, Nantes, France; Instituto de Biotecnologia/UNAM

**Keywords:** nebovirus, bovine calicivirus, attachment factor, HBGAs, binding spectrum, fucose

## Abstract

Some viruses within the Caliciviridae family initiate their replication cycle by attachment to cell surface carbohydrate moieties, histo-blood group antigens (HBGAs), and/or terminal sialic acids (SAs). Although bovine nebovirus (BNeV), one of the enteric caliciviruses, is an important causative agent of acute gastroenteritis in cattle, its attachment factors and possibly other cellular receptors remain unknown. Using a comprehensive series of protein-ligand biochemical assays, we sought to determine whether BNeV recognizes cell surface HBGAs and/or SAs as attachment factors. It was found that BNeV virus-like particles (VLPs) bound to A type/H type 2/Le^y^ HBGAs expressed in the bovine digestive tract and are related to HBGAs expressed in humans and other host species, suggesting a wide spectrum of HBGA recognition by BNeV. BNeV VLPs also bound to a large variety of different bovine and human saliva samples of all ABH and Lewis types, supporting previously obtained results and suggesting a zoonotic potential of BNeV transmission. Removal of α1,2-linked fucose and α1,3/4-linked fucose epitopes of target HBGAs by confirmation-specific enzymes reduced the binding of BNeV VLPs to synthetic HBGAs, bovine and human saliva, cultured cell lines, and bovine small intestine mucosa, further supporting a wide HBGA binding spectrum of BNeV through recognition of α1,2-linked fucose and α1,3/4-linked fucose epitopes of targeted HBGAs. However, removal of terminal α2,3- and α2,6-linked SAs by their specific enzyme had no inhibitory effects on binding of BNeV VLPs, indicating that BNeV does not use terminal SAs as attachment factors. Further details of the binding specificity of BNeV remain to be explored.

**IMPORTANCE** Enteric caliciviruses such as noroviruses, sapoviruses, and recoviruses are the most important etiological agents of severe acute gastroenteritis in humans and many other mammalian host species. They initiate infection by attachment to cell surface carbohydrate moieties, HBGAs, and/or terminal SAs. However, the attachment factor(s) for BNeV, a recently classified enteric calicivirus genus/type species, remains unexplored. Here, we demonstrate that BNeV VLPs have a wide spectrum of binding to synthetic HBGAs, bovine and human saliva samples, and bovine duodenal sections. We further discovered that α1,2-linked fucose and α1,3/4-linked fucose epitopes are essential for binding of BNeV VLPs. However, BNeV VLPs do not bind to terminal SAs on cell carbohydrates. Continued investigation regarding the proteinaceous receptor(s) will be necessary for better understanding of the tropism, pathogenesis, and host range of this important viral genus.

## INTRODUCTION

The binding of an infectious virus particle through attachment factors and receptors on the host cell surface is the essential first step for the viral entry and subsequent replication therein (1, 2). Generally, attachment factors facilitate the concentration of the incoming virus particles on the cell surface but do not actively promote entry and mediate signals, whereas receptors bind the viruses, promote entry, and activate cellular signaling pathways (3). Viral attachment factors and receptors on host cells comprise a large variety of proteins, carbohydrates, and lipids with physiological functions unrelated to pathogen interaction (3). For many viruses, these receptors are glycans linked to either a protein (glycoprotein or proteoglycan) or a glycolipid (4–6). Glycan-dependent viruses use glycoepitopes as receptors, binding to negatively charged sialic acids (SAs), sulfated oligosaccharide motifs of glycosaminoglycan (GAG) chains, or neutral glycoepitopes such as those found on histo-blood group antigens (HBGAs) (6). SAs at the termini or inner portions of glycan chains serve as receptors for at least 10 different virus families, whereas representatives from at least eight different virus families use GAG chains as receptors (4–7). In contrast, only a few human and animal viruses within a few families, including Caliciviridae, Parvoviridae, and Reoviridae, use HBGAs as receptors (2, 6, 8). The selection of different glycoepitopes as attachment factors may contribute to virus tropism, pathogenesis, and host specificity (5, 6, 8, 9).

Viruses within the family Caliciviridae are small, nonenveloped, icosahedral viruses that possess single-stranded, positive-sense genomic RNA of 7 to 8 kb in size (10). This family contains five established genera, Lagovirus, Nebovirus, Norovirus, Sapovirus, and Vesivirus (11). Recently, six additional unclassified caliciviruses may represent new genera, tentatively named Bavovirus (12, 13), Nacovirus (13–15), Recovirus (16), Salovirus (17), Sanovirus (18), and Valovirus (19). Caliciviruses are important etiologic agents in humans and animals, causing a variety of diseases in their respective hosts, such as respiratory disease (feline calicivirus [FCV]), hemorrhagic disease (rabbit hemorrhagic disease virus [RHDV]), and gastroenteritis (norovirus [NoV], sapovirus [SaV], and nebovirus [NeV]).

Several caliciviruses utilize cell surface carbohydrate moieties, SAs, or HBGAs as attachment factors (2). The initial observation that the Lagovirus RHDV uses the H type 2 HBGA as an attachment factor (20) inspired studies to identify similar factors for the other members of the Caliciviridae family (21). These studies showed that different HBGAs are used as attachment factors for human NoVs (HuNoVs) (21, 22), bovine NoV (23), canine NoVs (24), and primate enteric caliciviruses within the Recovirus genus (25). In contrast, FCV (26), murine NoV (MNV) (27), and porcine SaV (PSaV) (28) utilize terminal SAs as attachment factors. Recently, it was observed that some HuNoVs and monkey recoviruses also utilize SAs as attachment factors (29, 30). Finally, proteinaceous cellular surface structures were identified as receptors for a few caliciviruses, such as CD300lf and CD300ld for MNV (31, 32) and junctional adhesion molecule-1 (JAM-1) for FCV and Hom-1 calicivirus (33–35).

HBGAs are complex carbohydrates linked to glycoproteins or glycolipids found in red blood cells and epithelial cells of the gastrointestinal, genitourinary, and respiratory tracts in a wide variety of species (2, 8). They can also be secreted as free oligosaccharides into bodily fluids, such as saliva, intestinal content, milk, and blood (2, 8). The ABH and Lewis HBGAs are synthesized by the stepwise addition of monosaccharide units to five different types of precursor: type 1 (Galβ-3GlcNAcβ1-R), type 2 (Galβ-4GlcNAcβ1-R), type 3 (Galβ-3GalNAcα1-R), type 4 (Galβ-3GalNAcβ1-R), and type 5 (Galβ-4Glcβ1-Cer) (36). Each step is catalyzed by specific glycosyltransferases, such as α-1,2 fucosyltransferase (FUT2), α-1,3 or α-1,4 fucosyltransferase (FUT3), and two glycosyltransferases (A and B enzymes) (2, 8). For example, the α-1,2 fucosyltransferase adds a fucose residue at the α-1,2 linkage position of galactose, generating H antigen motifs (2, 8). The addition of *N*-acetylgalactosamine (GalNAc) or galactose at the α-1,3 position of H type chains via A enzyme or B enzyme results in either A or B antigen (2, 8, 24). The *FUT3* gene, as well as the *FUT4*, *FUT5*, *FUT6*, *FUT7*, and *FUT9* genes, generate the Lewis antigens by adding a fucose residue at either the α-1,3 or α-1,4 linkage position of the *N*-acetylglucosamine (GlcNAc) in the type 1 and/or type 2 precursors (2, 8, 24).

The genus Nebovirus contains one established type species, Newbury-1 virus, that contains Nebraska-like and Newbury-1-like clades (37, 38). Recently, two more species in the genus Nebovirus have been identified (39, 40). The reported fecal prevalence of the bovine nebovirus (BNeV) in calf diarrhea is 5% in Tunisia (41), 4.8% in Brazil (42), 7% in France (40), 9.1% in Korea (38), 13.1% in Italy (43), and 21.6% in the United States (44). Moreover, the BNeV prototype strains Newbury-1 and Nebraska experimentally induce diarrhea and small intestinal pathology, such as the desquamation of villous epithelial cells and villous atrophy in gnotobiotic calves (45–47). Despite its significant economic impact on the livestock industry and status as a pathogen with zoonotic potential, the BNeV life cycle remains largely unknown, mainly due to a lack of robust and reproducible *in vitro* cultivation systems. Based on the information that either SAs or HBGAs are used as attachment factors for many caliciviruses, we hypothesized that BNeV also recognizes either SAs or HBGAs as attachment factors for entry and infection. Therefore, the objective of this study was to determine the interaction between BNeV virus-like particles (VLPs) and either SAs or HBGAs using a comprehensive series of BNeV-ligand biochemical assays in synthetic HBGAs, bovine and human saliva samples, cell cultures, and bovine intestinal tissue sections.

## RESULTS

### Production and characteristics of BNeV VLPs and hyperimmune antiserum.

**FIG 1:**
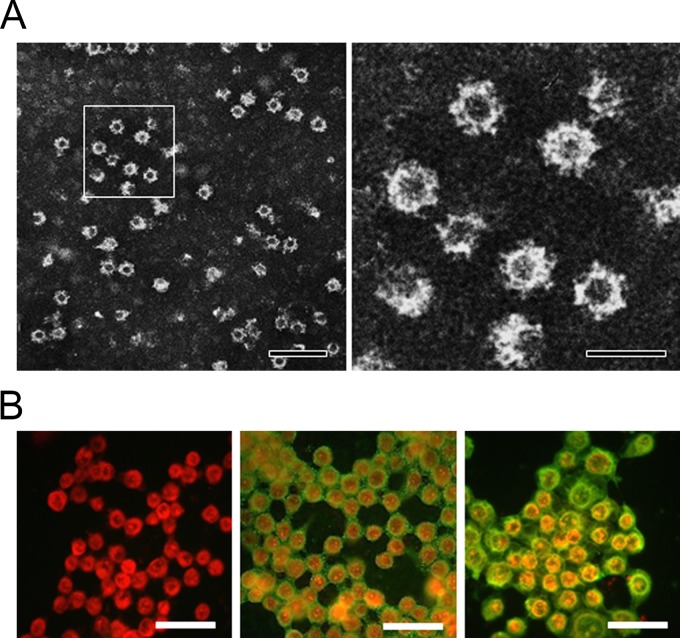
Electron micrograph of BNeV VLPs from and detection of BNeV capsid protein in rMA415-infected insect cells by immunofluorescence assay. (A) The rMA415 VLPs were purified by CsCl gradients from the cell culture supernatants of rMA415-infected Sf9 cells and visualized by negative staining with 3% phosphotungstic acid (pH 7.0). The panel on the right is a magnification of the panel on the left. The scale bars in the left and right panels correspond to 100 nm and 50 nm, respectively. (B) Sf9 cells were mock infected (left) or infected with rMA415. After 48 (middle) and 72 (right) h postinfection, cells were immunostained using the rabbit hyperimmune serum against BNeV capsid protein and FITC-conjugated goat anti-rabbit IgG antibody. The scale bars for left and right panels correspond to 50 μm.

The VLPs produced from the Spodoptera frugiperda ovarian (Sf9) cells infected with the recombinant baculovirus rMA415 had a size of 35 to 40 nm and appeared empty by electron microscopy (EM) due to the lack of viral nucleic acids (Fig. 1A). Hyperimmune antiserum generated from a rabbit immunized three times with purified VLPs of rMA415 by CsCl density gradient ultracentrifugation detected a specific signal by immunofluorescence in Sf9 cells infected with recombinant baculovirus rMA415 but not in wild-type baculovirus-infected SF9 cells (Fig. 1B). Western blotting with rMA415 hyperimmune antisera detected a 58-kDa protein as expected and consistent with previous reports on other caliciviruses (48, 49).

### Carbohydrate moieties act as attachment factors for BNeV.

**FIG 2:**
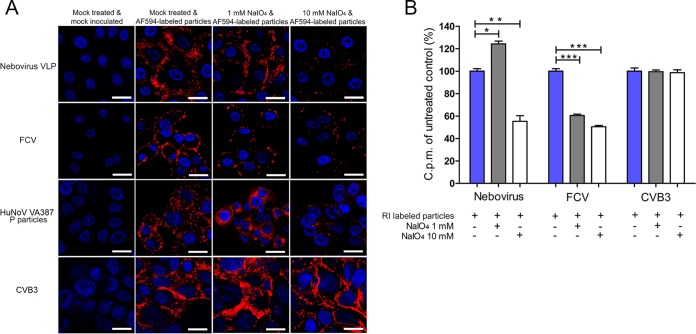
Binding of BNeV VLPs to cell surface carbohydrate. MDBK, CRFK, Caco-2, and HeLa cells were pretreated with 1 mM or 10 mM NaIO_4_ to remove the carbohydrate moieties. (A) Cells were incubated with the AF594-labeled BNeV VLPs and P particles of human NoV VA387 strain, FCV F9 strain, and CVB3 Nancy strain at 10 μg/ml and then examined by confocal microscopy. (B) The [^35^S]methionine/cysteine-labeled BNeV VLPs, control FCV F9 strain, or CVB3 Nancy strain (50,000 cpm) was bound to MDBK, CRFK, or HeLa cells following pretreatment with NaIO_4_ or no pretreatment. Binding was quantified by liquid scintillation counting. All experiments were performed three independent times, and panel A shows one representative set of results. The scale bars in panel A correspond to 10 μm. Error bars indicate standard deviations (SD) from triplicate samples. *, *P* < 0.05; **, *P* < 0.005.

To examine whether carbohydrate moieties are used as attachment factors for BNeV, the carbohydrate moieties were removed from MDBK cells by pretreatment with sodium periodate (NaIO_4_), which is known to remove the carbohydrate groups without altering cell surface proteins or membranes (50). The binding of Alexa Fluor 594 (AF594)-labeled BNeV VLPs was slightly but significantly increased by pretreating the MDBK cells with 1 mM NaIO_4_ but was markedly decreased with 10 mM NaIO_4_ (Fig. 2A). Pretreatment of cells with NaIO_4_ dose dependently decreased the binding of the SA-dependent FCV F9 strain, consistent with the high sensitivity of SA to periodate treatment (26). HBGA-dependent VLPs from the HuNoV strain VA387 showed a binding pattern similar to that of BNeV, indicative of binding to neutral sugars (51, 52). However, 1 or 10 mM NaIO_4_ pretreatment had no inhibitory effect on the binding of the coxsackievirus B3 (CVB3) strain Nancy, which is known to use decay-accelerating factor (DAF) as a cellular receptor (7, 53).

To precisely quantify the inhibitory effect of NaIO_4_ treatment, radioisotope (RI)-labeled BNeV VLPs and the FCV and CVB3 strains were incubated with cells that were not pretreated or were pretreated with NaIO_4_ (as mentioned above), and then the degree of binding was measured for each virus by liquid scintillation counting (28). As expected, binding of BNeV VLPs increased to 124% with the 1 mM NaIO_4_ treatment and decreased to 55% with the 10 mM NaIO_4_ treatment compared with the mock-treated, VLP-inoculated control (Fig. 2B). The binding of SA-dependent FCV was strongly decreased by both 1 and 10 mM NaIO_4_ treatment, whereas DAF-dependent CVB3 was not influenced by the treatment (Fig. 2B). Taken together, these results strongly suggest that neutral carbohydrate moieties are involved in the binding of BNeV VLPs to cells.

### Terminal SAs are not recognized by BNeV for attachment.

**FIG 3:**
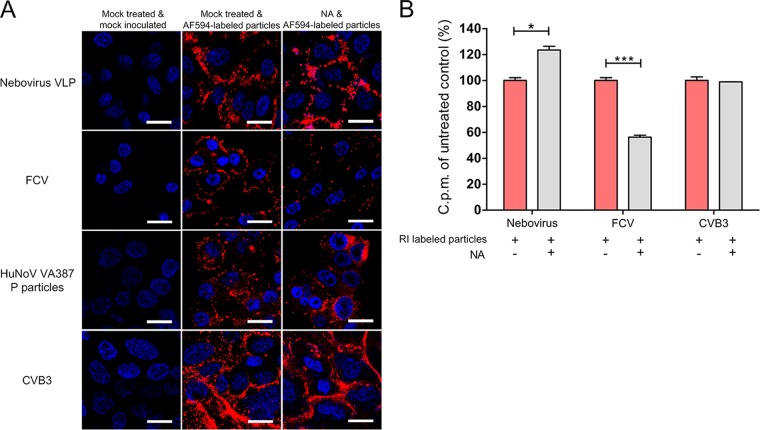
Lack of BNeV VLP binding to cell surface terminal sialic acids (SAs). MDBK, CRFK, Caco-2, and HeLa cells were pretreated with 100 mU V. cholerae neuraminidase (NA) ml^−1^ to remove α2,3-, α2,6-, and α2,8-linked SAs from the carbohydrate moieties. (A) The AF594-labeled BNeV VLPs, FCV F9 strain, and P particles of human NoV VA387 strain and CVB3 Nancy strain were added to the cells at 10 μg/ml and then examined by confocal microscopy. (B) The [^35^S]methionine/cysteine-labeled BNeV VLPs, control FCV F9 strain, or CVB3 Nancy strain (50,000 cpm) was bound to MDBK, CRFK, Caco-2, or HeLa cells after being pretreated with NA or with no pretreatment. Binding was measured by liquid scintillation counting. All experiments were performed three independent times, and panel A shows one representative set of results. The scale bars correspond to 10 μm. Error bars indicate SD determined from triplicate samples. *, *P* < 0.05; **, *P* < 0.005.

SAs represent a family of sugar molecules that are found mostly at the terminal end of carbohydrates and attach to underlying glycans via α2,3, α2,6, or α2,8 linkages (4). Because several caliciviruses, including FCV, PSaV, and MNV, use terminal SAs as attachment factors (5, 26–28), we examined whether SAs act as attachment factors for BNeV using 100 mU Vibrio cholerae neuraminidase (NA) ml^−1^, which cleaves α2,3-, α2,6-, and α2,8-linked SAs (26, 28). Pretreatment of MDBK cells with NA increased the binding of AF594-labeled BNeV VLPs to cells (Fig. 3A), and the binding of RI-labeled BNeV VLPs to cells increased to 123% of the levels observed in the mock-treated cells (Fig. 3B). A similar degree of enhanced binding was observed in the cells treated with HBGA-dependent HuNoV P particles (Fig. 3A). However, SA-dependent FCV showed a marked reduction in cell binding, whereas with DAF-dependent CVB3 the NA pretreatment had no influence on the degree of binding to cells (Fig. 3). These results support the notion that BNeV does not use terminal SAs as attachment factors and that it uses neutral carbohydrate motifs such as HBGAs instead.

### Wide binding spectrum of BNeV VLPs to HBGAs.

**FIG 4:**
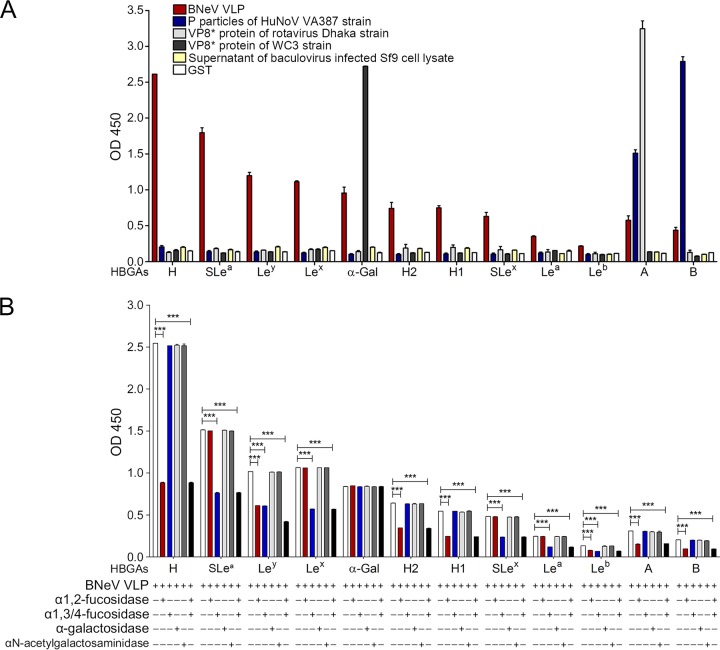
Binding and blocking of BNeV VLPs to synthetic HBGAs. (A) Ninety-six-well plates were coated with 50 μg/ml BNeV VLPs, GST-tagged-VP8* protein and GST-tagged P particles (10 μg/ml), supernatant of wild-type baculovirus-infected Sf9 cells lysate, and GST and then incubated with each of the synthetic HBGAs (10 μg/ml). The binding of each HBGA to target viral proteins and the control was determined by addition of horseradish peroxidase-conjugated streptavidin as described in Materials and Methods. (B) α1,2-Linked fucose, α1,3/4-linked fucose, αGal, and GalNAc epitopes were removed from each synthetic HBGA coated in each well using the corresponding enzyme. After incubation of BNeV VLPs at 50 μg/ml, the binding of BNeV VLPs was determined using hyperimmune serum against BNeV capsid protein, followed by addition of horseradish peroxidase-conjugated goat anti-rabbit IgG antibody. The signal intensities for graphs in panels A and B were visualized using TMB at 450 nm in three independent experiments. Error bars indicate SD from triplicate samples.

Using a synthetic HBGA binding assay, we next determined whether HBGAs could be used as attachment factors for BNeV, as has been described for other viruses elsewhere (28, 54–56). The BNeV VLPs strongly bound to the immobilized synthetic disaccharide (Fucα1,2Gal) (Fig. 4A), a common motif in HBGAs (57). Moreover, sialyl-Le^a^ (SLe^a^), Le^y^, Le^x^, αGal, H2, H1, SLe^x^, A, and B type immobilized synthetic HBGA oligosaccharides interacted with the BNeV VLPs (ordered from highest to lowest binding degree) (Fig. 4A). The recombinant glutathione *S*-transferase (GST)-P particles of the HuNoV strain VA387 and the GST-VP8* proteins of the human rotavirus G11P[25] Dhaka6 and bovine rotavirus G6P[5] WC3 strains bound to their corresponding HBGA types, whereas the supernatant of wild-type baculovirus-infected Sf9 cells and GST had no binding affinity to any HBGA (Fig. 4A). These results indicate that BNeV VLPs recognize a wide spectrum of HBGAs.

### HBGA-binding moiety for BNeV VLPs.

**FIG 5:**
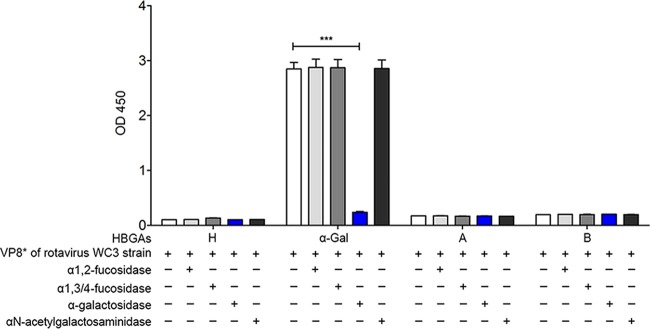
Blocking of the binding of GST-VP8* protein of bovine rotavirus P[5] WC3 strain to synthetic HBGAs. α1,2-linked fucose, α1,3/4-linked fucose, αGal, and GalNAc epitopes were removed from each synthetic HBGA using the corresponding enzyme. Reduction in binding specificity to the VP8* protein of the bovine rotavirus P[5] WC3 strain was determined using the HBGA-binding assay. The signal intensities were visualized by TMB at 450 nm in three independent experiments. Error bars indicate SD from triplicate samples.

To identify HBGA-binding epitopes, we examined whether removal of each putative epitope from synthetic HBGAs by pretreatment with α1,2-l-fucosidase, α1,3/4-l-fucosidase, α-galactosidase, or α-*N*-acetylgalactosaminidase was able to decrease the HBGA binding of BNeV VLPs. The results showed that treatment with α1,2-l-fucosidase, which removes the α-1,2-linked fucose from galactose, significantly decreased the binding of BNeV VLPs to the H type disaccharide, H1 and H2 types, and Le^y^ carrying an α1,2-linked fucose epitope (Fig. 4B), suggesting that BNeV VLPs recognize the α1,2-linked fucose as an epitope. In contrast, pretreatment with α1,3/4-l-fucosidase resulted in a significant reduction in the binding of BNeV VLPs to SLe^a^, Le^x^, SLe^x^, and Le^y^, which all harbor the α1,3/4-linked fucose epitope (Fig. 4B), confirming binding specificity for this epitope. An almost complete reduction in the binding of BNeV VLPs to Le^y^, containing both α1,2- and α1,3/4-linked fucose epitopes, was observed by combined pretreatment of synthetic Le^y^ with both enzymes, α1,2-l-fucosidase and α1,3/4-l-fucosidase, supporting the above-described results that BNeV VLPs recognize both α1,2-linked fucose and α1,3/4-linked fucose. Pretreatment of the Galα3Galβ4GlcNAcβ HBGA with α-galactosidase, which cleaves the αGal epitope in Galα3Galβ4GlcNAcβ HBGA, had no inhibitory effect on the binding of BNeV VLPs to synthetic Galα3Galβ4GlcNAcβ HBGA (Fig. 4B). However, it significantly reduced the binding of the control GST-VP8* protein of the αGal-dependent bovine rotavirus P[5] WC3 strain to the synthetic Galα3Galβ4GlcNAcβ HBGA (Fig. 5), suggesting that the αGal epitope is not recognized by the BNeV VLPs. Altogether, these results suggest that BNeV VLPs recognize fucose residues in α1,2 and α1,3/4 linkages.

### Saliva binding profile of BNeV VLPs.

**FIG 6:**
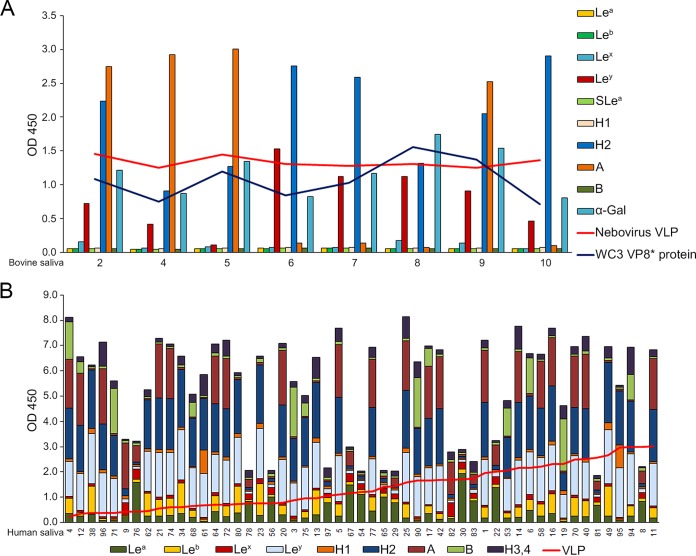
Binding between BNeV VLPs and saliva samples. (A) Expression levels of each HBGA in eight boiled bovine saliva samples coated onto 96-well plates were determined using mouse monoclonal antibodies specific for each HBGA, followed by the addition of horseradish peroxidase-conjugated goat anti-mouse IgG and IgM antibody. After characterization of HBGA expression levels in each saliva sample, binding specificity of BNeV VLPs and VP8* protein of bovine rotavirus strain WC3 to each saliva sample was determined by saliva-binding assay. The horizontal red line represents the OD values for BNeV VLP binding for each saliva sample. The horizontal blue line represents the OD values for VP8* protein of bovine rotavirus WC3 strain. (B) Expression levels of each HBGA in 53 human saliva samples were determined as described above. Binding specificity of BNeV VLPs to each saliva sample was determined by saliva-binding assay as described in Materials and Methods. The horizontal red line represents the OD values for BNeV VLP binding for each saliva sample. Binding of BNeV VLPs to each sample was visualized using TMB and measured at 450 nm in three independent experiments. Error bars indicate SD from triplicate samples.

Because saliva contains mucins carrying HBGAs that are similar to those expressed in the small intestine (2, 8), a saliva-binding assay was performed with the BNeV VLPs, the GST-P particles of the HuNoV strain VA387, and the GST-VP8* proteins of the bovine rotavirus P[5] strain WC3 and human rotavirus P[25] strain Dhaka6 using bovine and human saliva samples, as described elsewhere (55, 56, 58). Prior to determining the binding affinity of BNeV VLPs in the bovine and human saliva samples, the expression levels of each HBGA in the bovine and human saliva samples were examined by enzyme immunoassays as described elsewhere (51, 52, 56). Consistent with previous HBGA phenotyping results for bovine gastrointestinal mucosa (23), the bovine saliva samples contained individually various levels of A type, H type 2, Le^y^, and Galα3Galβ4GlcNAcβ HBGAs and were largely divided into two ABO blood types, H^+^/A^−^/B^−^ and H^+^/A^+^/B^−^ (Fig. 6A). In addition, the human saliva samples also had variable levels of HBGAs, in accordance with ABO and Lewis types (Fig. 6B).

**FIG 7:**
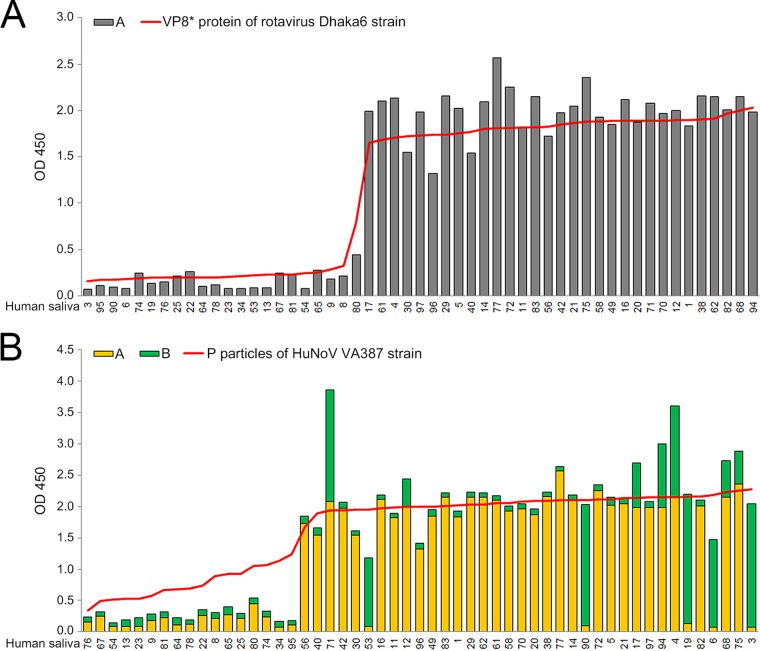
Binding of the rotavirus VP8* protein and human NoV P particles to human saliva samples. (A) The GST-VP8* protein of the human rotavirus P[25] Dhaka6 strain was tested as a positive control for binding to a panel of saliva samples from 53 human individuals. Binding results for the individual saliva samples were sorted by A type and non-A type HBGA signals for the individual saliva samples. (B) The GST-P particles of the human norovirus strain VA387 (GII.4) were tested as a positive control for binding to a panel of saliva samples from 53 human individuals. The binding of P particles was plotted by sorting of the A and B type signals from individual saliva samples. Binding of BNeV VLPs to each sample was visualized using TMB and measured at 450 nm in three independent experiments. Error bars indicate SD from triplicate samples.

Subsequently, the binding of BNeV VLPs to HBGAs in the bovine and human saliva samples was examined. The BNeV VLPs bound to HBGAs in the bovine saliva samples regardless of the contents of individual HBGAs (Fig. 6A). As a positive control, the recombinant GST-VP8* protein of the bovine rotavirus strain WC3 bound to bovine saliva samples (Fig. 6A). An analogous result was obtained for binding to a range of human saliva samples, regardless of the contents of individual HBGAs (Fig. 6B). As controls, the recombinant GST-VP8* protein of the human rotavirus strain Dhaka6 preferentially bound to human saliva samples rich in A type HBGA (Fig. 7A), whereas the recombinant GST-P particles of the HuNoV strain VA387 showed preferential binding to human saliva samples rich in A and B types of HBGAs (Fig. 7B). All of these data are consistent with the conclusion that a broad range of HBGAs is recognized by BNeV VLPs.

### Binding epitopes for BNeV VLPs in the saliva samples.

**FIG 8:**
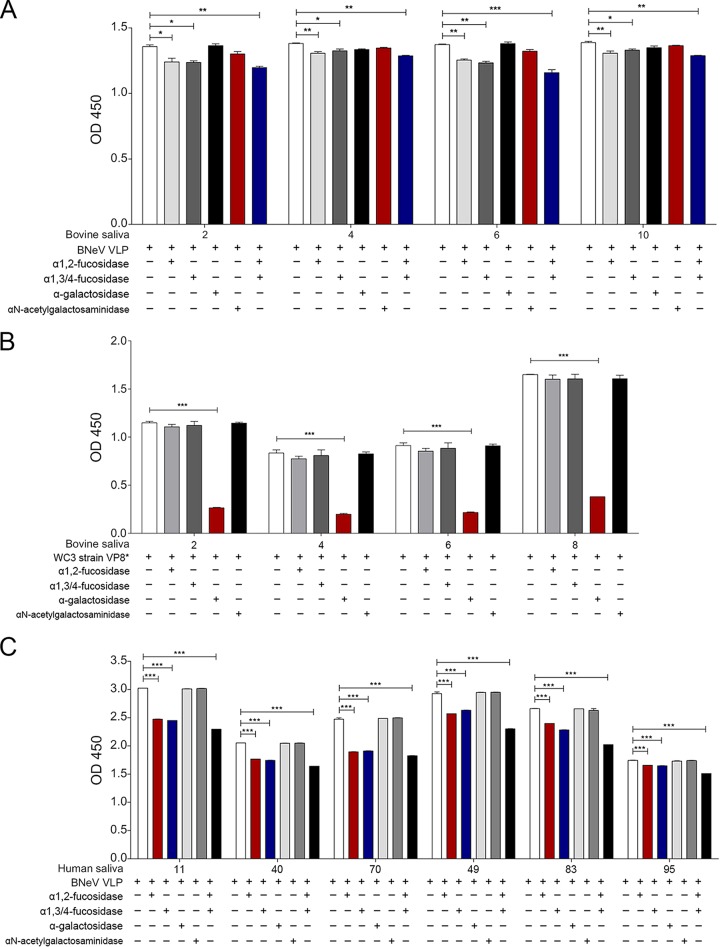
Blocking of binding of BNeV VLPs and VP8* protein of bovine P[5] strain WC3 to bovine and human saliva samples. (A) Four selected bovine saliva samples expressing either A type (H^+^/A^+^/B^−^) or H type (H^+^/A^−^/B^−^) were coated onto 96-well plates prior to removal of α1,2-linked fucose, α1,3/4-linked fucose, αGal, and GalNAc epitopes from HBGAs by pretreatment with a single specific enzyme or a combination of enzymes. A reduction in the HBGA binding specificity of BNeV VLPs to each bovine saliva sample was determined by saliva-binding assay using hyperimmune serum against BNeV capsid protein as described in Materials and Methods. (B) The GST-VP8* protein of the bovine rotavirus P[5] WC3 strain was used as a positive control. Four bovine saliva samples expressing either A type (H^+^/A^+^/B^−^) or H type (H^+^/A^−^/B^−^) HBGAs were used to remove α1,2-linked fucose, α1,3/4-linked fucose, αGal, and GalNAc epitopes from HBGAs in the saliva samples by individual or combinatorial enzyme pretreatment. A reduction in the HBGA binding specificity of VP8* protein of bovine rotavirus strain WC3 was determined by a saliva-binding assay using hyperimmune serum against VP8* protein. (C) Six human saliva samples expressing either A type (H^+^/A^+^/B^−^) or H type (H^+^/A^−^/B^−^) were used to remove α1,2-linked fucose, α1,3/4-linked fucose, αGal, and GalNAc epitopes from HBGAs in the saliva samples by pretreatment with each specific enzyme individually or in various combinations. Reduction in the HBGA binding specificity of BNeV VLPs was determined by a saliva-binding assay using hyperimmune serum against BNeV capsid protein as described in Materials and Methods. Blocking of BNeV VLPs to each sample was visualized using TMB and measured at 450 nm in three independent experiments. Error bars indicate SD from triplicate samples.

To identify binding epitopes in saliva HBGAs, the effect of pretreating the saliva samples with α1,2-l-fucosidase, α1,3/4-l-fucosidase, α-galactosidase, or α-*N*-acetylgalactosaminidase, either individually or in combination, was examined. Four bovine saliva samples were selected, two of which represented the H^+^/A^+^/B^−^ type (samples 2 and 4) and two of which represented the H^+^/A^−^/B^−^ type (samples 6 and 10). As each bovine saliva sample expressed different levels of A type, H type 2, Le^y^ type, and Galα3Galβ4GlcNAcβ, pretreatment with individual enzymes reduced the binding of BNeV to each saliva sample only mildly (Fig. 8A). However, pretreatment of saliva samples with a mixture of α1,2-l-fucosidase and α1,3/4-l-fucosidase resulted in greater inhibition (Fig. 8A). In addition, pretreatment of the bovine saliva samples with α-galactosidase and α-*N*-acetylgalactosaminidase did not inhibit the binding of BNeV VLPs to any of the selected bovine saliva samples (Fig. 8A), supporting the interpretation that the αGal and GalNAc epitopes are not recognized by the BNeV VLPs. To assess the function of α-galactosidase, the inhibitory effect of α-galactosidase on the binding of the GST-VP8* protein of αGal-dependent bovine rotavirus P[5] strain WC3 to each of the bovine saliva samples was examined. Pretreatment of the bovine saliva samples with α-galactosidase reduced the binding with the control GST-VP8* protein of the αGal-dependent bovine rotavirus P[5] strain WC3 (Fig. 8B).

Each human saliva sample used in this study had different ABH and Lewis antigens depending on the individual (Fig. 6B). Among these samples, six samples representing A (samples 11, 40, and 70) or H (samples 49, 83, and 95) ABH types were selected. Regardless of the ABH types, pretreatment of each saliva sample with a mixture of α1,2-l-fucosidase and α1,3/4-l-fucosidase inhibited the BNeV binding significantly more than that with either α1,2-l-fucosidase or α1,3/4-l-fucosidase individually (Fig. 8C). As expected, pretreatment of the human saliva samples with α-galactosidase or α-*N*-acetylgalactosaminidase had no influence on BNeV binding (Fig. 8C). These results confirmed the wide HBGA-binding spectrum of BNeV VLPs through recognition of α1,2-linked fucose and α1,3/4-linked fucose epitopes.

### Expression of BNeV-binding HBGA epitopes in cell lines.

**FIG 9:**
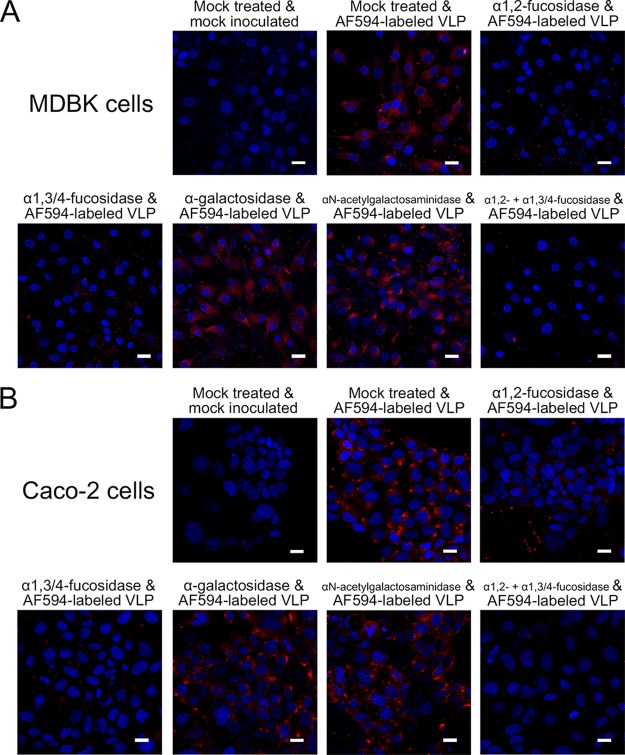
Determination of BNeV-binding HBGA epitopes in MDBK and Caco-2 cells. (A) Bovine kidney epithelial MDBK cells were pretreated with or without each specific enzyme (α1,2-l-fucosidase, α1,3/4-l-fucosidase, α-galactosidase, or α-*N*-acetylgalactosaminidase) individually or in various combinations as indicated, mock treated, or applied with 10 μg/ml AF594-labeled BNeV VLPs and then examined by confocal microscopy. (B) To remove α1,2-linked fucose, α1,3/4-linked fucose, αGal, GalNAc epitopes, or α1,2- and α1,3/4-linked fucoses together, human colorectal adenocarcinoma Caco-2 cells were pretreated with or without each individual enzyme or combinations of the enzymes as indicated, mock treated, or applied with AF594-labeled BNeV VLPs at 10 μg/ml and then examined by confocal microscopy. All experiments were performed three independent times, and panels A to C show one representative set of results. The scale bars correspond to 50 μm.

To further define the BNeV-binding HBGA epitopes, the expression levels of the different HBGAs were examined in several cell lines using antibodies specific for each HBGA. The bovine kidney epithelial MDBK, porcine kidney epithelial LLC-PK, canine kidney epithelial MDCK, and feline kidney CRFK cells solely expressed Galα3Galβ4GlcNAcβ HBGA. Human colorectal adenocarcinoma Caco-2 cells expressed H types 1 and 2, Le^a^, Le^x^, Le^b^, and Le^y^ HBGAs, while human embryonic kidney epithelial 293T cells did not express any of the HBGAs examined. Among these cell lines, MDBK cells were selected to check whether αGal is recognized as an attachment factor for BNeV binding because of sole expression of the αGal epitope carrying Galα3Galβ4GlcNAcβ HBGA on the cell surface, whereas Caco-2 cells were selected to check whether α1,2-fucose and α1,3/4-fucose epitopes are used as BNeV attachment factors because of expression of multiple HBGAs containing α1,2-fucose and α1,3/4-fucose epitopes on the cell surface HBGAs. The binding of AF594-labeled BNeV VLPs to each cell line was then examined both before and after removal of all of the corresponding HBGAs expressed in each cell line by pretreatment with α1,2-l-fucosidase, α1,3/4-l-fucosidase, α-galactosidase, or α-*N*-acetylgalactosaminidase, either individually or in combination. As expected, removal of αGal from Galα3Galβ4GlcNAcβ HBGA by pretreatment with α-galactosidase had no inhibitory effect on BNeV binding to the MDBK cells, supporting the conclusion that Galα3Galβ4GlcNAcβ HBGA is not used for BNeV attachment (Fig. 9A). To confirm the efficacy of α1,2-l-fucosidase, α1,3/4-l-fucosidase, and α-galactosidase, MDBK and Caco-2 cells were pretreated with each enzyme and then the samples were checked for efficient removal of fucose by using *Ulex Europaeus* agglutinin 1 (UEA-1), which detects fucose residues (59), or an antibody specific to αGal. The pretreatment of cells with α-galactosidase markedly removed αGal residue from MDBK cells, whereas a mixture of α1,2-l-fucosidase and α1,3/4-l-fucosidase significantly removed fucose residues from Caco-2 cells (data not shown). Interestingly, pretreatment of MDBK cells with each fucosidase significantly decreased BNeV VLP binding (Fig. 9A). These effects became more apparent when the cells were pretreated with the mixture (Fig. 9A), even though the cells did not appear to express fucosylated HBGAs. This suggests that α1,2- and α1,3/4-linked fucose residues not detected by these reagents nonetheless support the binding of BNeV VLPs to MDBK cells. Pretreatment of Caco-2 cells with either α1,2-l-fucosidase or α1,3/4-l-fucosidase also reduced the binding of BNeV VLPs (Fig. 9B). The reduction was much enhanced by pretreatment of the Caco-2 cells with the α1,2-l-fucosidase and α1,3/4-l-fucosidase mixture (Fig. 9B), indicating that BNeV VLP binding involved the α1,2-linked fucose and α1,3/4-linked fucose epitopes of HBGAs.

### BNeV attachment to CHO cells with expression of H type 2.

**FIG 10:**
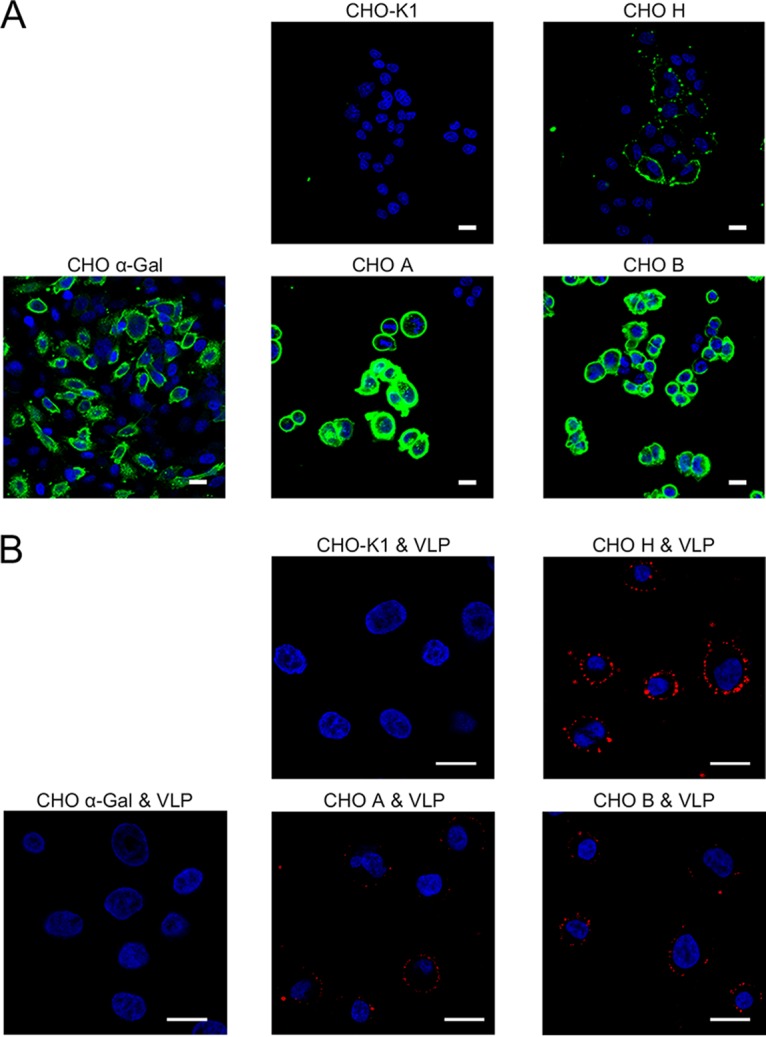
Binding of BNeV VLPs to parent and transfectant CHO cells stably expressing HBGAs. (A) The expression of target HBGA in the parental CHO (H^−^/A^−^/B^−^) cells, single-transfectant CHO cells expressing the H antigen (H^+^/A^−^/B^−^) or the αGal antigen (H^−^/A^−^/B^−^/αGal^+^), and double-transfectant CHO cells expressing either the A antigen (H^+^/A^+^/B^−^) or the B antigen (H^+^/A^−^/B^+^) were determined using antibodies specific for each target HBGA via confocal microscopy. (B) The parental and transfectant CHO cells stably expressing target HBGA were applied with AF594-labeld BNeV VLPs (10 μg/ml) and then examined by confocal microscopy. All experiments were performed three independent times, and each panel shows one representative set of results. The scale bars correspond to 50 μm.

CHO cells do not express any HBGA on the cell surface due to the lack of α1,2-fucosyltransferase activity and of either the A or B histo-blood group enzymes (60). Whether the above-described binding between BNeV VLPs and HBGAs was similar to that found in parental CHO or transfectant CHO cells, parent and transfectant CHO cells expressing H type 2, A type, B type, or Galα3Galβ4GlcNAcβ HBGAs were examined. After the expression of each target HBGA in parental CHO and transfectant CHO cells, as confirmed (Fig. 10A), binding of BNeV VLPs to parental and transfectant CHO cells was analyzed. Compared with that of parental CHO cells (H^−^/A^−^/B^−^), to which AF594-labeled BNeV VLPs failed to attach, binding of BNeV VLPs was very prominent in CHO cells expressing H type 2 HBGA (H^+^/A^−^/B^−^) (Fig. 10B). It was also detected, albeit less extensively, with A (H^+^/A^+^/B^−^) and B (H^+^/A^−^/B^+^) types (Fig. 10B). As expected, CHO cells expressing Galα3Galβ4GlcNAcβ HBGA had no BNeV VLP binding (Fig. 10B). Taken together, these findings support the overall conclusion that BNeV VLPs have a wide HBGA binding spectrum through specific reorganization of α1,2- and α1,3/4-linked fucose residues in the HBGAs.

### Determination of BNeV-binding HBGA epitopes in bovine duodenal epithelium.

The above-described results showed that the BNeV VLPs had no binding specificity for terminal SAs on the cultured cell surface. To confirm these results in bovine intestinal tissues, whether removal of terminal or internal parts of cell surface carbohydrate moieties using 1 or 10 mM NaIO_4_ pretreatment could influence the attachment of BNeV to bovine duodenal epithelium was examined (23). Similar to the aforementioned findings (Fig. 2), abolishment of BNeV binding to the duodenal epithelium was achieved with the 10 mM but not the 1 mM NaIO_4_ pretreatment (data not shown), suggesting that neutral sugars are also involved in binding to gut tissue. Consistent with this finding, removal of α2,3- and α2,6-linked SAs using NA from V. cholerae failed to inhibit BNeV VLP binding to gut epithelium (data not shown).

We previously demonstrated by immunohistochemistry that bovine duodenal epithelium expresses A type, H type 2, Le^y^, and αGal HBGAs but no other HBGAs (23). Confirming our previous results (23), bovine duodenal sections had two ABO blood types, H^+^/A^−^/B^−^/Ley^+^/α-Gal^+^ and H^+^/A^+^/B^−^/Ley^+^/α-Gal^+^ (data not shown). These results are consistent with the above-described findings for the HBGA phenotypes in the saliva samples, with A and O blood types being present in cattle (Fig. 6).

**FIG 11:**
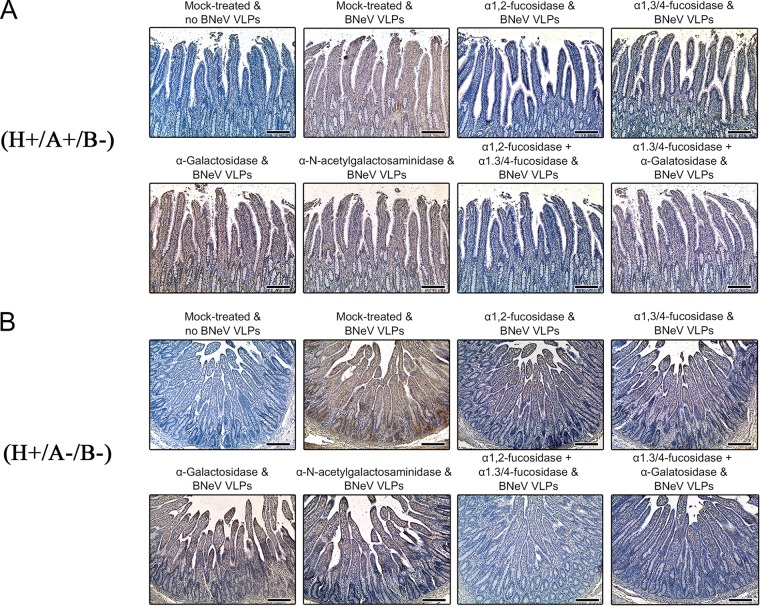
Binding inhibition of BNeV VLPs to bovine small intestinal epithelium by HBGA epitope-specific enzymes. H^+^/A^+^/B^−^/Le^y+^/α-Gal^+^ tissue sections (A) and H^+^/A^−^/B^−^/Le^y+^/α-Gal^+^ tissue sections (B) were pretreated with enzymes specifically cleaving α-1,2-linked fucose, α-1,3/4-linked fucose, αGal, or GalNAc prior to incubation with BNeV VLPs at 10 μg/ml. Binding of BNeV VLPs was then detected using a rabbit hyperimmune serum against BNeV capsid protein. The experiment was performed in triplicate, and one representative set of results is shown. The scale bars correspond to 200 μm.

To determine whether BNeV VLPs can recognize HBGAs by binding to α1,2-linked fucose, α1,3/4-linked fucose, αGal, or GalNAc epitopes of HBGAs, bovine duodenal paraffin sections showing either O blood type (H^+^/A^−^/B^−^/Le^y+^/α-Gal^+^) or A blood type (H^+^/A^+^/B^−^/Le^y+^/α-Gal^+^) were selected and then pretreated with α1,2-l-fucosidase, α1,3/4-l-fucosidase, α-galactosidase, or α-*N*-acetylgalactosaminidase either individually or in dual combination. As found for the inhibitory effects of HBGA epitope-specific enzymes against BNeV binding to synthetic HBGAs (Fig. 4B), bovine and human saliva samples (Fig. 8), or cultured cells (Fig. 9), pretreatment of O or A blood type duodenal sections with α-galactosidase or α-*N*-acetylgalactosaminidase produced no inhibitory BNeV binding effects (Fig. 11). However, pretreatment of these duodenal sections with α1,2-l-fucosidase significantly reduced BNeV VLP binding. Pretreatment of duodenal sections with α1,3/4-l-fucosidase partially inhibited the binding of BNeV VLPs to the duodenal epithelium, while pretreatment of the duodenal sections with a combination of α1,2-l-fucosidase and α1,3/4-l-fucosidase almost completely abolished the binding of BNeV VLPs to the duodenal epithelium (Fig. 11). Taken together, these results indicate that BNeV VLPs can attach to bovine as well as human glycan compounds on the surface of epithelial cells containing α1,2-linked fucose and α1,3/4-linked fucose epitopes, as found in most HBGA epitopes.

## DISCUSSION

Viruses must attach to cell surface attachment factor(s) and/or receptor(s) to initiate viral entry and replication (1, 2). Many caliciviruses attach to cell surface carbohydrate moieties, such as HBGAs or SAs (2, 28). Here, we demonstrate that VLPs of the BNeV bind to a wide spectrum of HBGAs present as synthetic neoglycoconjugates, secreted in bovine and human saliva, or expressed by cultured cells and bovine duodenal epithelium. Among HBGA-dependent caliciviruses, some HuNoVs, particularly some GII.4 strains and the recently emerged GII.17 strains, are well known to have a wide binding spectrum of HBGAs in all ABO, Lewis, and secretor/nonsecretor types (2, 58, 61, 62). Generally, it is considered that the multiple binding patterns of HuNoVs to HBGAs could be subdivided into two major binding groups: the A/B or Lewis binding groups (2, 51, 52). The A/B binding group members, including VA387, Norwalk, and MOH strains, are considered to accommodate one or two epitopes of A/B and H HBGAs, i.e., galactose and/or α1,2-linked fucose. In contrast, the Lewis binding group members, such as the Boxer, VA207, and OIF strains, utilize α1,2-linked fucose and/or α1,3-linked fucose epitopes of H and Lewis HBGAs. In the present study, enzymatic removal of α1,2- and α1,3/4-linked fucoses from the various HBGA backbones reduced BNeV binding to the corresponding synthetic HBGAs and saliva samples. These data indicate that similar to the human Lewis binding group, BNeV VLPs recognize a wide spectrum of HBGAs via binding to their α1,2- and α1,3/4-linked fucose epitopes (2, 51, 52, 58, 61). As expected, removal of the αGal or GalNAc epitopes had no inhibitory effect on BNeV VLP binding, indicating that BNeV does not use the αGal and GalNAc epitopes as attachment factors. Indeed, it is well known that bovine genogroup III NoV uses the αGal epitope, which is absent from human and pig gut epithelium, suggesting that neither humans nor pigs could be infected by bovine NoVs (23). However, this remains to be explored.

The binding of BNeV VLPs to the bovine duodenal sections was markedly decreased by pretreatment with α1,2-l-fucosidase. This was attributed to the A type/H type 2/Le^y^ HBGAs from the four types being expressed in bovine duodenal epithelium containing the α1,2-linked fucose epitope. In addition, there is a slight reduction in BNeV binding to the bovine duodenal sections with the α1,3/4-l-fucosidase pretreatment because the α1,3/4-linked fucose epitope is present only in Le^y^ HBGA. However, pretreatment of bovine duodenal sections with α-galactosidase or α-*N*-acetylgalactosaminidase had no inhibitory effect on the binding of BNeV VLPs to the duodenal sections, indicating that the αGal epitope in Galα3Galβ4GlcNAcβ HBGA and the GalNAc epitope in A type HBGA were not used as attachment epitopes for BNeV binding to the bovine duodenum. These findings also support the conclusion that similar to the human Lewis binding group, BNeV VLPs attach to α1,2- and/or α1,3/4-linked fucose epitopes in H type 2/Le^y^ HBGAs expressed in the bovine duodenal epithelium (2, 51, 52, 58, 61).

As demonstrated in the present study, BNeV had a wide HBGA binding spectrum through recognition of α1,2-linked fucose and α1,3/4-linked fucose epitopes of targeted HBGAs. It should be noted that bovine and human saliva samples have different levels of ABH and Lewis antigens which carry α1,2-linked fucose and/or α1,3/4-linked fucose epitopes (23). Therefore, pretreatment of bovine and human saliva samples with a mixture of α1,2-l-fucosidase and α1,3/4-l-fucosidase should have greater inhibitory effects on BNeV binding than that with either α1,2-l-fucosidase or α1,3/4-l-fucosidase individually.

Although the synthetic HBGA binding assay showed that the VLPs from BNeV had strong binding to SLe^a^ HBGA and weak binding to SLe^x^ HBGA, they were expressed in the bovine small intestinal epithelium or secreted into saliva with the methods used. This means that BNeV does not use SLe^a^ and SLe^x^ for entry and infection in cattle. Both SLe^a^ and SLe^x^ are expressed at high concentrations in cancer cells such as human colon cancer but only minimally in nontransformed cells, defining their utility as diagnostic cancer markers in human medicine (63). The reason for the binding of BNeV VLPs to SLe^a^ and SLe^x^ is the presence of α1,3/4-linked fucose epitopes (57).

SA-containing gangliosides have been identified as attachment factors for murine MNV1, PSaV, and FCV (26–28). Interestingly, recent studies have shown that in addition to HBGAs, SAs also can be used as an attachment factor(s) for HuNoVs and Tulane virus, the prototype of the Recovirus genus; α2,3- and α2,6-linked SAs, particularly α2,3-linked SA containing GM3, possibly act as attachment factors for HuNoV VA387 (GII.4) and VA115 (GI.3) strains, whereas α2,6-linked terminal SAs are likely utilized by Tulane virus (29, 30). In contrast to the reduced binding observed for the Tulane virus (29), in this study, removal of terminal SAs from cell surface carbohydrates by NA and 1 mM NaIO_4_ had no inhibitory effect on the binding of BNeV VLPs to the cells. Rather, pretreatment with NA and 1 mM NaIO_4_ increased BNeV VLP binding. This may be due to increased access to fucosylated epitopes following removal of the charged SA motifs. However, pretreatment with 10 mM NaIO_4_ markedly decreased the BNeV binding to the cells, possibly due to complete removal of HBGAs on the cell surface (23). The lack of inhibition following NA treatment does not rule out the possibility that the internal SAs of gangliosides like GM1a are recognized (64). However, the lack of sensitivity with the 1 mM NaIO_4_ treatment strongly suggests that this is not the case.

In the present study, we demonstrate that BNeV VLPs do not recognize the αGal epitope. Additionally, MDBK cells were found to solely express the αGal epitope and not to express fucosylated HBGA with the antibodies used. Therefore, it was anticipated that α1,2- and α1,3/4-linked fucose epitope-dependent BNeV VLPs would not bind the cell surface of MDBK cells. Unexpectedly, however, BNeV VLPs attached to MDBK cells and pretreatment of MDBK cells with these fucosidases inhibited BNeV binding to MDBK cells. The mechanism by which BNeV VLPs bind to MDBK cells remains unclear. Nevertheless, these data suggest that MDBK cells express fucosylated HBGAs, which could not be detected by the antibodies used in this study but could be degraded by fucosidases. The identification of specific fucose-containing epitopes on MDBK cells involved in BNeV binding forms the basis of an ongoing work.

Because infection by all viruses begins with the attachment of the virus to the host cells, expression levels of the target receptor for a given virus could be an important factor in determining the viral tropisms, pathogenesis, and host range restriction (6, 65–67). Some NoVs detected in humans and animals have a close genetic relationship and share target receptors, which has raised questions regarding the possibility of the zoonotic transmission of these viruses (24, 68). For example, porcine NoV strains detected worldwide have close genetic relatedness to HuNoVs (69–76). Moreover, HuNoV strain GII.4-HS66 induces diarrhea and intestinal pathology in piglets and calves, respectively (77, 78). Recent studies have shown that primate enteric caliciviruses within the Recovirus genus share their HBGA attachment factors with HuNoVs (25, 79). Furthermore, the zoonotic potential of these viruses has been clearly demonstrated by the high prevalence of neutralizing antibodies (80, 81). In the present study, BNeV utilized HBGAs that are commonly used by HuNoVs and other enteric caliciviruses. This suggests that BNeVs have the potential to infect humans and/or other species. In particular, VLPs from BNeV displayed wide-spectrum binding in human saliva and human and animal cell lines, further supporting the above-described hypothesis. It should be noted that bovines express type 2-based HBGAs in their digestive tract but humans have type 1-based structures in their digestive tract. Accordingly, HuNoVs appear to favor the recognition of type 1-based HBGAs (82, 83). BNeVs might be less adapted to human infection, as neither the virus nor its specific antibody have been isolated from and detected in humans.

To demonstrate the direct interspecies transmission of BNeVs, the molecular detection of BNeVs and sequence analysis of resultant amplicons are necessary in stool samples of humans and animals such as pigs, particularly where humans and animals or different animal species live in close physical contact and mixed infections are more common (81). Although robust and reproducible *in vitro* cultivation systems for BNeVs have not been established, more definitive evidence for the interspecies transmission of BNeVs can be obtained by the inoculation of the BNeV isolates into different species, such as human volunteers or piglets. Indirect evidence for interspecies transmission of BNeVs can be provided by the detection of antibodies against BNeVs in serum samples collected from humans and other animals, particularly those living in the above-described environments (80, 84, 85). Nevertheless, our results stress the need for more in-depth genomic and serological studies of BNeVs in humans and other species.

In conclusion, the present study provides direct evidence that BNeV VLPs attach to H type 2/Le^y^/Le^x^ HBGAs expressed in the bovine digestive tract through their α1,2- and α1,3/4-linked fucose residues. Moreover, the usage of multiple HBGAs by BNeV VLPs and their ability to bind to human saliva suggest that BNeVs have the potential for zoonotic transmission. More in-depth epidemiological studies using human fecal and serum samples are required to determine the zoonotic potential of BNeVs. Similarly, continued investigations regarding the proteinaceous receptor(s) are necessary for a better understanding of the tropism, pathogenesis, and host range of this important viral genus.

## MATERIALS AND METHODS

### Cells and viruses.

Madin-Darby bovine kidney (MDBK), porcine kidney LLC-PK, and human cervical cancer HeLa cells purchased from the American Type Culture Collection (ATCC) were maintained in Eagle's minimum essential medium supplemented with 10% fetal bovine serum (FBS), 100 U/ml penicillin, and 100 μg/ml streptomycin as described elsewhere (7, 86). Madin-Darby canine kidney (MDCK), Crandell-Reese feline kidney (CRFK), human embryonic kidney 293T (HEK293T), and human colorectal adenocarcinoma Caco-2 cells, purchased from the ATCC, were grown in Dulbecco's modified Eagle's medium (DMEM) supplemented with 5% FBS, 100 U/ml penicillin, and 100 μg/ml streptomycin (7, 85). The parental Chinese hamster ovary (CHO) cells that do not express HBGAs (H^−^/A^−^/B^−^) were maintained in RPMI 1640 supplemented with 10% FBS, 1% l-glutamine, 100 U/ml penicillin, 100 μg/ml streptomycin, and 10 μg/ml each of adenosine, 2-deoxy-adenosine, and thymidine (60). In addition, single-transfectant CHO cells expressing the H antigen (H^+^/A^−^/B^−^) or the αGal antigen and double-transfectant CHO cells expressing either the A antigen (H^+^/A^+^/B^−^) or the B antigen (H^+^/A^−^/B^+^) were cultured under the conditions described for the parental CHO cells, with addition of 0.2 mg/ml hygromycin and 0.25 mg/ml of G418 (neomycin) to maintain the plasmids expressing the glycosylation enzymes (60). Sf9 cells, purchased from Gibco (Fort Worth, TX), were cultured at 27°C in SF-900 II SFM medium containing 10% FBS, 100 U/ml penicillin, 100 μg/ml streptomycin, lipid medium supplement, and 0.1% pluronic acid solution (Sigma-Aldrich, St. Louis, MO, USA). The FCV F9 strain (ATCC) and CVB3 Nancy strain were propagated in CRFK cells and HeLa cells, respectively (26, 87). Cesium chloride (CsCl) density gradient ultracentrifugation was used to purify each mass-cultured strain as described elsewhere (7).

### Reagents and antibodies.

NaIO_4_ and NA, from Sigma-Aldrich, were dissolved in phosphate-buffered saline (PBS; pH 7.2). α1,2-l-Fucosidase from Corynebacterium (TaKaRa Bio Inc., Kyoto, Japan), α1,3/4-l-fucosidase from Streptomyces (TaKaRa Bio Inc.), α-galactosidase from Coffea arabica (Sigma-Aldrich), α-*N*-acetylgalactosaminidase from Chryseobacterium meningosepticum (New England BioLabs, Inc., MA, USA), and UEA-1 (Vector Laboratories, Burlingame, CA, USA) were diluted in PBS. AF594 succinimidyl ester, purchased from Molecular Probes (Eugene, OR, USA), was dissolved in dimethyl sulfoxide (DMSO). [^35^S]methionine/cysteine was purchased from PerkinElmer (Waltham, MA). Biotin-conjugated oligosaccharides, including Lewis antigens (Le^a^, Le^b^, Le^x^, and Le^y^), H type, type A disaccharide, type B disaccharide, type A trisaccharide, type B trisaccharide, αGal trisaccharide, sialyl-Le^a^ (SLe^a^), and SLe^x^ tetrasaccharides, were purchased from GlycoTech (Gaithersburg, MD) (7). The following antibodies were used in this study: hyperimmune sera against BNeV capsid protein, P particles of human NoV VA387 strain, and VP8* protein of human rotavirus Wa strain, generated from rabbits by serial inoculation with each target protein as described below, monoclonal antibodies (MAbs) of anti-GST (Santa Cruz Biotechnology), anti-blood group A type antigen (types 1 and 2 chains) (Covance, NJ, USA), anti-blood group H antigen (type 1 chain) (Covance), anti-Le^a^ antigen (type 1 chain) (Covance), anti-Le^b^ antigen (type 1 chain) (Covance), anti-Le^x^ antigen (type 2 chain) (Covance), anti-Le^y^ antigen (type 2 chain) (Covance), anti-blood group B antigen (Thermo Scientific, MA, USA), anti-H type 2 antigen (Thermo Scientific), anti-αGal epitope antigen (Enzo Life Sciences, Seoul, South Korea), anti-glyceraldehyde-3-phosphate dehydrogenase (GAPDH) MAb (Santa Cruz Biotechnology), anti-rabbit IgG-fluorescence isothiocyanate (FITC)-conjugated antibody (Jackson ImmunoResearch Lab, West Grove, PA, USA), biotinylated goat anti-mouse or anti-rabbit antibodies (Dako, Glostrup, Denmark), and anti-mouse IgG-FITC-conjugated antibody (Santa Cruz Biotechnology). Horseradish peroxidase (HRP)-conjugated streptavidin and HRP-conjugated goat anti-rabbit immunoglobulin G (IgG) and anti-mouse IgG antibodies were obtained from Jackson ImmunoResearch Laboratories or Dako.

### Treatment of cells with chemicals and enzymes.

To determine whether BNeVs recognize terminal SAs as attachment factors, the following methods were used as described previously (7). Cells were treated with 1 or 10 mM NaIO_4_ for 30 min at 4°C or with NA at 100 mU for 1 h at 37°C in PBS. After the pretreatment, cells were washed three times with PBS. The binding assays were then carried out as described below. Mock and control treatments were performed at the same time.

### Expression and purification of BNeV VLPs.

BNeV VLPs were generated from a calf diarrhea fecal sample determined to be positive for BNeV by a PCR-based method (38) as described elsewhere (48, 49). Briefly, the complete 2.3-kb capsid region (encoding the VP1-major capsid and VP2-minor capsid regions) of Bo/BNeV/MA415/04/KR was amplified from the above-described fecal sample by reverse transcription-PCR (RT-PCR) with forward (5′-AAACATGAGTGACAACAAAAGCATCCCAGA-3′, nucleotide position 5055 to 5084 of VP1 region) and reverse (5′-TCAAACACTCGTGGTCGAGAACACTGAC-3′, nucleotide position 7360 to 7387 of VP2 region) primers designed from the full-length sequence of Newbury agent 1 strain in the GenBank database (accession number NC_007916). The amplicon was ligated into the pCR2.1-TOPO vector (Invitrogen, CA) and then transformed to DH5α competent cells (Enzynomics, Daejeon, South Korea). Plasmids were purified using GeneAll Hybrid-Q Plasmid Rapidpre (GeneAll, Seoul, South Korea), and the sequence (GenBank accession numbers EF528565 and MG009451) was verified using an ABI system 3700 automated DNA sequencer (Applied Biosystems, Foster City, CA). Using purified plasmid, the full-length cDNA copy of capsid gene was amplified by PCR with a forward primer (5′-CACAGGATCCATGAGTGACAACAAAAGCAT-3′) containing a BamHI restriction site (underlined) and reverse primer (5′-AATCTCGAGTCAAACACTCGTGGTCG-3′) containing an XhoI restriction site (underlined). After digestion with BamHI and XhoI restriction enzymes, the amplified fragments were subcloned into pFastBac1 baculovirus donor plasmid (ThermoFisher Scientific, Seoul, South Korea). The pFastBac1 donor plasmid was transformed into DH10Bac Escherichia coli, and its resultant recombinant bacmid DNA was transfected into Sf9 cells using Cellfectin II reagent (by following the manufacturer's instructions; Invitrogen). BNeV VLPs were expressed in baculovirus recombinant-transformed Sf9 insect cells at 27°C and harvested at 5 to 7 days postinfection. The cloned recombinant baculovirus generated from pFastBac1 plasmid containing VP1 and VP2 regions of MA415 strain was designated rMA415 and propagated in Sf9 cells to make master virus stocks. BNeV VLPs were purified using CsCl density gradient ultracentrifugation as described elsewhere (7). The protein concentrations of the VLPs were determined with a bicinchoninic acid (BCA) protein assay kit (Pierce, IL, USA) according to the manufacturer's instructions. Expression of recombinant capsid protein was validated by electron microscopy, immunofluorescence, and Western blot analyses as described elsewhere (48, 49, 88).

### Electron microscopy.

VLPs purified from rMA415-infected Sf9 cell culture supernatants by CsCl density gradient ultracentrifugation were stained with 3% phosphotungstic acid (pH 7.0) and examined with an electron microscope (JEM-2000 FXII; JEOL, USA) as described previously (49).

### Production of rabbit hyperimmune antiserum.

A rabbit hyperimmune antiserum against BNeV VLPs, P particles of human NoV VA387 strain, and VP8* protein of human rotavirus Wa strain was produced as described elsewhere (48, 49). Briefly, two rabbits for each target viral protein were subcutaneously immunized three times with purified BNeV VLPs, P particles of HuNoV, or VP8* protein of rotavirus in complete Freund's adjuvant for the first injection or incomplete Freund's adjuvant for the subsequent infections. The animals were bled 2 weeks after the last booster injection.

### Coomassie blue staining and Western blot analysis.

To check the quality and expression of BNeV VLPs, Coomassie blue staining and Western blot analysis were performed as described elsewhere (48, 49, 88). Briefly, the proteins in the supernatant of cells infected with rMA415 or wild-type baculovirus were concentrated by precipitation with 8% polyethylene glycol. The cells infected with rMA415 or wild-type baculovirus were washed three times with cold PBS and lysed using cell extraction buffer containing 10 mM Tris-HCl, pH 7.4, 100 mM NaCl, 1 mM EDTA, 1 mM EGTA, 1 mM NaF, 20 mM Na_2_P_2_O_7_, 2 mM Na_3_VO_4_, 1% Triton X-100, 10% glycerol, 0.1% SDS, and 0.5% deoxycholate (Invitrogen) for 30 min on ice. Lysates were spun down by centrifugation at 12,000 × *g* for 10 min at 4°C, and the samples were analyzed for total protein content with a BCA protein assay kit (Thermo Scientific, Waltham, MA, USA). Samples were resolved by SDS-PAGE and served for Coomassie blue staining or transferred onto nitrocellulose membranes (GE Healthcare Life Sciences). The membranes were blocked for 1 h at room temperature with Tris-buffered saline containing 5% skimmed milk before they were incubated overnight at 4°C with the primary rabbit polyclonal antibody against BNeV capsid protein. The bound antibody was developed by incubation with an HRP-labeled secondary antibody, and the immunoreactive bands were detected by enhanced chemiluminescence (ECL) (Dogen, Seoul, South Korea) using a Davinch-K imaging system (Youngwha Scientific Co., Ltd., Seoul, South Korea).

### Expression and purification of the GST-P particle and GST-VP8* protein.

The GST-P particles of the HuNoV strain VA387 (GII.4) and the GST-VP8* proteins of the human rotavirus P[25] Dhaka6 and bovine rotavirus P[5] WC3 strains were cloned, expressed, and purified as described previously (55, 89). The concentrations of the purified NoV P particles and VP8* proteins of the rotavirus strains were determined using a BCA protein assay kit (Pierce, IL, USA) according to the manufacturer's instructions.

### AF594 labeling of viruses and VLPs.

The FCV F9 and CVB3 Nancy strains and BNeV VLPs, purified by CsCl density gradient ultracentrifugation, were labeled with AF594 as described previously (7). Briefly, purified virus particles and VLPs (10 mg at 1 mg ml^−1^) in 0.1 M sodium bicarbonate buffer (pH 8.3) were labeled with a one-tenth-fold molar concentration of AF594 succinimidyl ester (1 mg at 1 mg ml^−1^ in DMSO). After thorough vortexing for 30 s, each mixture was incubated for 1 h at room temperature with continuous stirring. Labeled viruses and VLPs were repurified by CsCl density gradient ultracentrifugation, dialyzed, and stored in 2-μg aliquots at −20°C (90). The concentrations of the purified AF594-labeled BNeV VLPs, FCV strain, and CVB3 strain were determined using a BCA protein assay kit (Pierce, IL, USA) according to the manufacturer's instructions. Analysis of SDS-PAGE-separated, AF594-labeled viral particles and VLPs using Coomassie blue staining and Western blotting showed that the label was exclusively coupled to each viral protein.

### Immunofluorescence assay.

To determine the expression levels of each HBGA antigen on the cell surface, the binding specificity of AF594-labeled BNeV VLPs, FCV strain, and CVB3 strain to various cell lines, including transfectant CHO cells, as well as the expression levels of BNeV VLPs in the Sf9 cells infected with rMA415, the immunofluorescence assay was performed as described elsewhere (86, 88). Briefly, the confluent cells grown on eight-chamber slides were left untreated or were treated with chemicals or enzymes and then fixed with 4% paraformaldehyde in PBS for 1 h. For detection of the BNeV capsid proteins in the Sf9 cells, mock- or rMA415-infected Sf9 cells grown on microscope cover slides were harvested at 48 and 72 h postinfection and then fixed with 4% paraformaldehyde in PBS for 1 h. The cells were then permeabilized by the addition of 0.2% Triton X-100 and washed with PBS containing 0.1% newborn calf serum (PBS-NCS). MAbs specific for each HBGA and a polyclonal antibody against BNeV capsid protein were added to each chamber or cover slide, and the slides were incubated at 4°C overnight. The cells were then washed three times with PBS-NCS, and FITC-conjugated secondary antibodies were added. After washing, the cells were treated with 4′,6-diamidino-2-phenylindole (DAPI) solution for the staining of nuclei, mounted using SlowFade Gold antifade reagent (Invitrogen), and then examined under an EZ-C1 confocal microscope using EZ-C1 software (Nikon, Japan). Another set of eight-chamber slides treated as described above was added with AF594-labeled BNeV VLPs and was used to observe binding as described above. For detecting the BNeV capsid protein in the infected Sf9 cells, mock- or rMA415-infected Sf9 cells were incubated with FITC-conjugated secondary antibody. After washing, the cells were mounted with 60% glycerol in PBS (pH 8.0) and then examined under a fluorescence microscope.

### Labeling of viruses and VLPs with [^35^S]methionine/cysteine.

Radioisotope labeling of the FCV F9 and CVB3 Nancy strains with [^35^S]methionine/cysteine (PerkinElmer) was carried out as described elsewhere (7, 26). Briefly, each individual virus was inoculated at a multiplicity of infection (MOI) of 0.1 PFU/cell into confluent monolayers of cells and incubated for 4 h at 37°C. The medium was replaced with RPMI 1640 lacking methionine and cysteine (Sigma-Aldrich). Cells were starved for 2 h and then supplemented with 1 Mbq [^35^S]methionine/cysteine ml^−1^ (PerkinElmer). At 72 h postinfection, each labeled virus was purified by CsCl density gradient ultracentrifugation as described previously (7). BNeV VLPs metabolically radiolabeled with [^35^S]methionine/cysteine (PerkinElmer) were prepared as described previously, with slight modifications (91). Briefly, Sf9 cells were infected with recombinant baculovirus at an MOI of 10 PFU per cell and then incubated for 28 h. The medium was replaced with nonsupplemented Grace's insect medium (Gibco). Cells were starved for 30 min, and then 30 μCi [^35^S]methionine/cysteine ml^−1^ (PerkinElmer) was added. At 4 to 6 h following radioisotope labeling, the medium was volumetrically replaced with the same amount of Grace's insect medium with Sf-900 II SFM (Gibco). The cultures were harvested when 80% of the cells showed cytopathic effects. Radioisotope-labeled VLPs were purified by CsCl density gradient ultracentrifugation as described previously (7). The concentrations of the purified labeled BNeV VLPs, FCV strain, and CVB3 strain were determined using a BCA protein assay kit (Pierce) according to the manufacturer's instructions.

### Attachment assay with [^35^S]methionine/cysteine-labeled VLPs and viruses.

Binding of ^35^[S]methionine/cysteine-labeled BNeV VLPs, and the FCV F9 and CVB3 Nancy strains to each corresponding cell line was performed as described elsewhere (7). Briefly, cells (4 × 10^4^/well) were plated into 96-well microtiter plates and then independently incubated with purified [^35^S]methionine/cysteine-labeled BNeV VLPs and the FCV and CVB3 strains (50,000 cpm) for 45 min on ice. Cells were washed three times with ice-cold PBS followed by cell lysis with 0.1% sodium dodecyl sulfate and 0.1 M NaOH. Total radioactivity in the cell lysate was determined by liquid scintillation counting.

### Synthetic HBGA binding assay.

To determine the binding specificity of BNeV VLPs, the GST-VP8* proteins of the human rotavirus P[25] strain Dhaka6 and the GST-P particles of the HuNoV strain VA387 to each HBGA, a synthetic oligosaccharide-based HBGA binding assay was carried out as described elsewhere (7). Briefly, 96-well microtiter plates were coated with 50 μg/ml BNeV VLPs, 10 μg/ml of each GST-tagged-VP8* protein and GST-tagged P particles, the supernatant of wild-type baculovirus-infected Sf9 cell lysate, and GST and then incubated at 4°C overnight. Coated plates were blocked with 5% bovine serum albumin (BSA) for 1 h at room temperature, and each synthetic oligosaccharide-polyacrylamide (PAA)-biotin conjugate (10 μg/ml) was then added and further incubated at 4°C overnight. Bound oligosaccharides were detected using HRP-conjugated streptavidin. The signal intensities were visualized by 3,3′,5,5′-tetramethylbenzidine (TMB; Komabiotech), and the absorbance was read at 450 nm in a plate reader. For each step, the plates were incubated at 37°C for 1 h and washed five times with PBS containing 0.05% Tween 20 (PBS-Tween 20).

### Determination of binding epitopes in each synthetic HBGA.

To determine the target HBGA epitopes for the BNeV VLPs, removal of each epitope from the synthetic HBGAs was performed as described previously, with slight modifications (23, 92, 93). Briefly, 96-well plates were coated with each of the synthetic HBGAs and incubated at 4°C for 6 h. The plates were washed thrice with PBS-Tween 20 and blocked with PBS-BSA. After washing three times with PBS, the coated plates were incubated with 100 μl solution containing 20 mU/ml of α-1,2-l-fucosidase, 10 μU/ml α-1,3/4-l-fucosidase, 3 mU/ml α-galactosidase, or 5 mU/ml α-*N*-acetylgalactosaminidase for 24 h at 37°C. Thereafter, the plates were washed thrice with PBS and incubated with 50 μg/ml of BNeV VLPs at 4°C for 1 h. After washing with PBS, the plates were incubated with a hyperimmune serum against BNeV capsid protein. Following this, the plates were washed thrice with PBS and treated with HRP-conjugated goat anti-rabbit IgG. The signals were visualized by TMB followed by absorbance measurement at 450 nm using a plate reader.

### Saliva binding assay.

Saliva samples from 53 human individuals and 8 cows were selected from the Archives of the Saliva Registry of the Laboratory of Veterinary Pathology, College of Veterinary Medicine, Chonnam National University, Gwangju, South Korea. Before performing the saliva binding assay, the amount of each HBGA in saliva samples was determined by enzyme immunoassays as described previously (51, 52, 56). Briefly, boiled saliva samples were diluted to 1:20 in PBS and then coated onto microtiter immunoplates (Thermo Fisher Scientific) at 4°C overnight. After blocking with PBS containing 5% BSA at 37°C for 1 h, MAbs specific to H1, H2, Le^a^, Le^b^, Le^x^, Le^y^, type A, type B, and αGal HBGAs were added to each well, and the plates were incubated for 1 h at 37°C. After washing, HRP-conjugated goat anti-mouse anti-IgG or IgM was added to each well. After each step, the plates were washed five times with PBS. The color reaction after substrate addition was measured as described above.

Binding of the BNeV VLPs, GST-P particles of the HuNoV strain VA387, GST-VP8* proteins of the human rotavirus P[25] strain Dhaka6, and GST-VP8* proteins of the bovine rotavirus P[5] strain WC3 was assessed using the saliva binding assay as described previously, with slight modifications (51, 52, 56). Briefly, boiled saliva samples were diluted to 1:20 and then coated onto 96-well plates at 4°C overnight. After blocking with PBS-BSA at 37°C for 1 h, 50 μg/ml of the BNeV VLPs and 10 μg/ml of each viral protein were added, followed by incubation for 1 h at 37°C. The bound target proteins were detected using an anti-GST antibody or hyperimmune serum against BNeV capsid protein diluted to 1:1,000, followed by addition of HRP-conjugated goat anti-mouse or anti-rabbit IgG antibody. The signal intensities after addition of substrate were displayed by a TMB kit as described above.

### Determination of HBGA binding epitopes in each saliva sample.

To determine the target HBGA epitope for BNeV VLPs, removal of epitopes from selected bovine and human saliva samples was performed as described previously, with slight modifications (23, 24, 93, 94). Briefly, boiled saliva samples were diluted to 1:20 and then coated onto 96-well plates at 4°C overnight. The plates were washed thrice with PBS-Tween 20 and then incubated with 100 μl solution containing 10 mU/ml of α1,2-l-fucosidase, 10 μU/ml of α1,3/4-l-fucosidase, 4 mU/ml of α-galactosidase, or 8 mU/ml of α-*N*-acetylgalactosaminidase for 48 h at 37°C. After blocking with PBS-BSA at 37°C for 1 h, 50 μg/ml BNeV VLPs was added and incubated for 1 h at 37°C. The bound target proteins were detected using a rabbit hyperimmune serum against BNeV capsid protein diluted to 1:1,000, followed by addition of HRP-conjugated goat anti-rabbit IgG antibody. The signal intensities after substrate addition were obtained using a TMB kit as described above.

### Tissue samples and immunohistochemical analysis.

Paraffin-embedded bovine small intestinal samples obtained by necropsy from healthy calves were selected from the Archives of the Tissue Registry of the Laboratory of Veterinary Pathology, College of Veterinary Medicine, Chonnam National University, Gwangju, South Korea. To determine the binding and inhibitory effects of NaIO_4_ and the enzymes on the binding of BNeV VLPs to bovine small intestinal villous epithelial cells, immunohistochemical analysis was performed as described elsewhere (23, 28). Briefly, tissue sections of 3-μm thickness were deparaffinized, rehydrated through a graded ethanol series, and washed in PBS. Thereafter, the sections were treated with 0.3% H_2_O_2_ in methanol for 20 min to quench endogenous peroxidase, washed three times with PBS, and blocked with PBS-BSA for 30 min at room temperature in a humid atmosphere to inhibit nonspecific binding. To determine the expression of HBGAs, duodenal sections were first treated with the primary MAbs specific for each HBGA and left at 4°C overnight. Washed sections were also either left untreated or treated with NaIO_4_ at 1 mM or 10 mM in 50 mM sodium acetate buffer (pH 5.0) for 30 min at room temperature, followed by a 10-min incubation with 1% glycine in PBS to remove terminal or internal cell surface carbohydrate moieties or with enzymes for 18 h at 37°C for removal of each HBGA epitope or cleaving terminal SAs from epithelial cells. After washing with PBS, the sections were incubated with 10 μg/ml BNeV VLPs, diluted in PBS-BSA, and left at 4°C overnight. They were then washed thrice with PBS-BSA and incubated with a rabbit hyperimmune serum against BNeV capsid protein at 4°C overnight. After washing with PBS, the sections were incubated with biotinylated goat anti-rabbit or anti-mouse secondary antibodies (Dako) followed by peroxidase-conjugated streptavidin (Dako). The reactions were developed with 3,3′-diaminobenzidine tetrahydrochloride (DAB; Vector Laboratories) followed by treatment with Mayer's hematoxylin solution (Merck, Germany) for counterstaining.

### Ethics statement.

All animals were handled in strict accordance with good animal practices, as described in the NIH *Guide for the Care and Use of Laboratory Animals* (95). The protocol was approved by the Committee on Ethics of Animal Experiments, CNU (permit number CNU IACUC-YB-2016-65). The human saliva samples, collected with written consent from volunteers, were handled in strict accordance with human subject protocols, as described in the guidance for the care and use of human samples of the CNU adhered from the WMA Declaration of Helsinki (*Ethical Principles for Medical Research Involving Human Subjects*) (96). The protocol was approved by the Committee for Research Ethics Concerning Human Subjects, CNU (CNU IBR no. 1040198-130807-BR-002-02).

### Statistical analyses and software.

Statistical analyses were performed on triplicate experiments using GraphPad Prism software, version 5.03 (GraphPad Software Inc., La Jolla, CA, USA), and a one-way analysis of variance test. *P* values of less than 0.05 were considered statistically significant. Figures were generated using Adobe Photoshop CS3 and Prism 5, version 5.03.

### Accession number(s).

The sequence for the VP2-minor capsid region of Korean BNeV strain MA415 (Bo/BNeV/MA415/04/KR) has been deposited in GenBank under accession number MG009451.1.

## References

[1] Grove J, Marsh M 2011 The cell biology of receptor-mediated virus entry. J Cell Biol 195:1071–1082. doi:10.1083/jcb.201108131. 22123832 PMC3246895

[2] Tan M, Jiang X 2014 Histo-blood group antigens: a common niche for norovirus and rotavirus. Expert Rev Mol Med 16:e5. doi:10.1017/erm.2014.2. 24606759 PMC12406300

[3] Helenius A 2013 Virus entry and uncoating, p 87–104. *In* Knipe DM, Howley PM, Cohen JI, Griffin DE, Lamb RA, Martin MA, Racaniello VR, Roizman B (ed), Fields virology, 6th ed Lippincott Williams & Wilkins, Philadelphia, PA.

[4] Chen X, Varki A 2010 Advances in the biology and chemistry of sialic acids. ACS Chem Biol 5:163–176. doi:10.1021/cb900266r. 20020717 PMC2825284

[5] Neu U, Bauer J, Stehle T 2011 Viruses and sialic acids: rules of engagement. Curr Opin Struct Biol 21:610–618. doi:10.1016/j.sbi.2011.08.009. 21917445 PMC3189341

[6] Olofsson S, Bergström T 2005 Glycoconjugate glycans as viral receptors. Ann Med 37:154–172. doi:10.1080/07853890510007340. 16019714

[7] Kim DS, Son KY, Koo KM, Kim JY, Alfajaro MM, Park JG, Hosmillo M, Soliman M, Baek YB, Cho EH, Lee JH, Kang MI, Goodfellow I, Cho KO 2016 Porcine sapelovirus uses α2,3-linked sialic acid on GD1a ganglioside as a receptor. J Virol 90:4067–4077. doi:10.1128/JVI.02449-15. 26865725 PMC4810533

[8] Le Pendu J, Nyström K, Ruvoën-Clouet N 2014 Host-pathogen co-evolution and glycan interactions. Curr Opin Virol 7:88–94. doi:10.1016/j.coviro.2014.06.001. 25000207

[9] Stehle T, Khan ZM 2014 Rules and exceptions: sialic acid variants and their role in determining viral tropism. J Virol 88:7696–7699. doi:10.1128/JVI.03683-13. 24807712 PMC4097780

[10] Green KY 2007 Caliciviridae: the noroviruses, p 582–608. *In* Knipe DM, Howley PM, Griffin DE, Lamb RA, Martin MA, Roizman B, Straus SE (ed), Fields virology, 5th ed Lippincott Williams & Wilkins, Philadelphia, PA.

[11] Clarke IN, Estes MK, Green KY, Hansman GS, Knowles NJ, Koopmans MK, Matson DO, Meyers G, Neill JD, Radford A, Smith AW, Studdert MJ, Thiel HJ, Vinjé J 2012 Caliciviridae, p 977–986. *In* King AMQ, Adams MJ, Carstens EB, Lefkowitz EJ (ed), Virus taxonomy. Classification and nomenclature of viruses. Ninth report of the International Committee on Taxonomy of Viruses Elsevier Academic Press, San Diego, CA.

[12] Wolf S, Reetz J, Otto P 2011 Genetic characterization of a novel calicivirus from a chicken. Arch Virol 156:1143–1150. doi:10.1007/s00705-011-0964-5. 21404111

[13] Wolf S, Reetz J, Hoffmann K, Gründel A, Schwarz BH, Hünel I, Otto P 2012 Discovery and genetic characterization of novel caliciviruses in German and Dutch poultry. Arch Virol 157:1499–1507. doi:10.1007/s00705-012-1326-7. 22580496

[14] Day JM, Ballard LL, Duke MV, Schefflerm BE, Zsak L 2010 Metagenomic analysis of the turkey gut RNA virus community. Virol J 7:313. doi:10.1186/1743-422X-7-313. 21073719 PMC2991317

[15] Liao Q, Wang X, Wang D, Zhang D 2014 Complete genome sequence of a novel calicivirus from a goose. Arch Virol 159:2529–2531. doi:10.1007/s00705-014-2083-6. 24756346

[16] Farkas T, Sestak K, Wei C, Jiang X 2008 Characterization of a rhesus monkey calicivirus representing a new genus of Caliciviridae. J Virol 82:5408–5416. doi:10.1128/JVI.00070-08. 18385231 PMC2395209

[17] Mikalsen AB, Nilsen P, Frøystad-Saugen M, Lindmo K, Eliassen TM, Rode M, Evensen O 2014 Characterization of a novel calicivirus causing systemic infection in Atlantic salmon (Salmo salar L.): proposal for a new genus of Caliciviridae. PLoS One 9:e107132. doi:10.1371/journal.pone.0107132. 25203050 PMC4159302

[18] Wang F, Wang M, Dong Y, Zhang B, Zhang D 2017 Genetic characterization of a novel calicivirus from a goose. Arch Virol 162:2115–2118. doi:10.1007/s00705-017-3302-8. 28289976

[19] L'Homme Y, Sansregret R, Plante-Fortier E, Lamontagne AM, Ouardani M, Lacroix G, Simard C 2009 Genomic characterization of swine caliciviruses representing a new genus of Caliciviridae. Virus Genes 39:66–75. doi:10.1007/s11262-009-0360-3. 19396587

[20] Ruvoën-Clouet N, Ganière JP, André-Fontaine G, Blanchard D, LePendu J 2000 Binding of rabbit hemorrhagic disease virus to antigens of the ABH histo-blood group family. J Virol 74:11950–11954. doi:10.1128/JVI.74.24.11950-11954.2000. 11090195 PMC112478

[21] Tan M, Jiang X 2010 Norovirus gastroenteritis, carbohydrate receptors, and animal models. PLoS Pathog 6:e10000983. doi:10.1371/journal.ppat.1000983. PMC2928792 20865168

[22] Marionneau S, Ruvoën N, Le Moullac-Vaidye B, Clement M, Cailleau-Thomas A, Ruiz-Palacois G, Huang P, Jing X, Le Pendu J 2002 Norwalk virus binds to histo-blood group antigens present on gastroduodenal epithelial cells of secretor individuals. Gastroenterology 122:1967–1977. doi:10.1053/gast.2002.33661. 12055602 PMC7172544

[23] Zakhour M, Ruvoën-Clouet N, Charpilienne A, Langpap B, Poncet D, Peters T, Bovin N, Le Pendu J 2009 The alphaGal epitope of the histo-blood group antigen family is a ligand for bovine norovirus Newbury2 expected to prevent cross-species transmission. PLoS Pathog 5:e1000504. doi:10.1371/journal.ppat.1000504. 19578439 PMC2699481

[24] Caddy S, Breiman A, Le Pendu J, Goodfellow I 2014 Genogroup IV and VI canine noroviruses interact with histo-blood group antigens. J Virol 88:10377–10391. doi:10.1128/JVI.01008-14. 25008923 PMC4178834

[25] Farkas T, Cross RW, Hargitt E III, Lerche NW, Morrow AL, Sestak K 2010 Genetic diversity and histo-blood group antigen interactions of rhesus enteric caliciviruses. J Virol 84:8617–8625. doi:10.1128/JVI.00630-10. 20554772 PMC2919043

[26] Stuart AD, Brown TD 2007 Alpha2,6-linked sialic acid acts as a receptor for feline calicivirus. J Gen Virol 88:177–186. doi:10.1099/vir.0.82158-0. 17170450

[27] Taube S, Perry JW, Yetming K, Patel SP, Auble H, Shu L, Nawar HF, Lee CH, Connell TD, Shayman JA, Wobus CE 2009 Gangloside-linked terminal sialic acid moieties on murine macrophages function as attachment receptors for murine noroviruses (MNV). J Virol 83:4092–4101. doi:10.1128/JVI.02245-08. 19244326 PMC2668497

[28] Kim DS, Hosmillo M, Alfajaro MM, Kim JY, Park JG, Son KY, Ryu EH, Sorgeloos F, Kwon HJ, Park SJ, Lee WS, Cho D, Kwon J, Choi JS, Kang MI, Goodfellow I, Cho KO 2014 Both α2,3- and 2,6-linked sialic acids on O-linked glycoproteins act as functional receptors for porcine sapovirus. PLoS Pathog 10:e1004172. doi:10.1371/journal.ppat.1004172. 24901849 PMC4047124

[29] Han L, Tan M, Xia M, Kitova EN, Jiang X, Klassen JS 2014 Gangliosides are ligands for human noroviruses. Am Chem Soc 136:12631–12637. doi:10.1021/ja505272n. PMC4160279 25105447

[30] Tan M, Wei C, Huang P, Fan Q, Quigley C, Xia M, Fang H, Zhang X, Zhong W, Klassen JS, Jiang X 2015 Tulane virus recognizes sialic acids as cellular receptors. Sci Rep 5:11784. doi:10.1038/srep11784. 26146020 PMC4491846

[31] Haga K, Fujimoto A, Takai-Todaka R, Miki M, Doan YH, Murakami K, Yokoyama M, Murata K, Nakanishi A, Katayama K 2016 Functional receptor molecules CD300lf and CD300ld within the CD300 family enable murine noroviruses to infect cells. Proc Natl Acad Sci U S A 113:E6248–E6255. doi:10.1073/pnas.1605575113. 27681626 PMC5068309

[32] Orchard RC, Wilen CB, Doench JG, Baldridge MT, McCune BT, Lee YCJ, Lee S, Pruett-Miller SM, Nelson CA, Fremont DH, Virgin HW 2016 Discovery of a proteinaceous cellular receptor for a norovirus. Science 353:933–936. doi:10.1126/science.aaf1220. 27540007 PMC5484048

[33] Bhella D, Goodfellow IG 2011 The cryo-electron microscopy structure of feline calicivurs bound to junctional adhesion molecule A at 9-angstrom resolution reveals receptor-induced flexibility and two distinct conformational changes in the capsid protein VP1. J Virol 85:11381–11390. doi:10.1128/JVI.05621-11. 21865392 PMC3194967

[34] Makino A, Shimojima M, Miyazawa T, Kato K, Tohya Y, Akashi H 2006 Junctional adhesion molecule 1 is a functional receptor for feline calicivirus. J Virol 80:4482–4490. doi:10.1128/JVI.80.9.4482-4490.2006. 16611908 PMC1472022

[35] Sosnovtsev SV, Sandoval-Jaime C, Parra GI, Tin CM, Jones RW, Soden J, Barnes D, Freeth J, Smith AW, Green KY 2017 Identification of human junctional adhesion molecules 1 as a functional receptor for the Hom-1 calicivirus on human cells. mBio 8:e00031-17. doi:10.1128/mBio.00031-17. 28196955 PMC5312078

[36] Ravn V, Dabelsteen E 2000 Tissue distribution of histo-blood group antigens. APMIS 108:1–28. doi:10.1034/j.1600-0463.2000.d01-1.x. 10698081

[37] Oliver SL, Asobayire E, Dastjerdi AM, Bridger JC 2006 Genomic characterization of the unclassified bovine enteric virus Newbury agent-1 (Newbury1) endorses a new genus in the family Caliciviridae. Virology 350:240–250. doi:10.1016/j.virol.2006.02.027. 16574184 PMC7111791

[38] Park SI, Jeong C, Park SU, Kim HH, Jeong YJ, Hyun BH, Chun YH, Kang MI, Cho KO 2008 Molecular detection and characterization of unclassified bovine enteric caliciviruses in South Korea. Vet Microbiol 130:371–379. doi:10.1016/j.vetmic.2008.01.017. 18387758 PMC7126893

[39] Alkan F, Karayel İ Catella C, Bodnar L, Lanave G, Bányai K, Di Martino B, Decaro N, Buonavoglia C, Martella V 2015 Identification of a bovine enteric calicivirus, Kırklareli virus, distantly related to neboviruses, in calves with enteritis in Turkey. J Clin Microbiol 53:3614–3617. doi:10.1128/JCM.01736-15. 26292294 PMC4609679

[40] Kaplon J, Guenau E, Asdrubal P, Pothier P, Ambert-Balay K 2011 Possible novel Nebovirus genotype in cattle, France. Emerg Infect Dis 17:1120–1123. doi:10.3201/eid/1706.100038. 21749786 PMC3358183

[41] Hassine-Zaafrane M, Kaplon J, Sdiri-Loulizi K, Aouni Z, Pothier P, Aouni M, Ambert-Balay K 2012 Molecular prevalence of bovine noroviruses and neboviruses detected in central-eastern Tunisia. Arch Virol 157:1599–1604. doi:10.1007/s00705-012-1344-5. 22585047

[42] Candido M, Alencar AL, Almeida-Queiroz SR, Buzinaro MG, Munin FS, Godoy SH, Livonesi MC, Fernandes AM, Sousa RL 2016 First detection and molecular characterization of nebovirus in Brazil. Epidemiol Infect 144:1876–1878. doi:10.1017/S0950268816000029. 26796080 PMC9150621

[43] Di Martino B, Di Profio F, Martella V, Ceci C, Marsilio F 2011 Evidence for recombination in neboviruses. Vet Microbiol 153:367–372. doi:10.1016/j.vetmic.2011.05.034. 21665387

[44] Cho YI, Han JI, Wang C, Cooper V, Schwartz K, Engelken T, Yoon KJ 2013 Case-control study of microbiological etiology associated with calf diarrhea. Vet Microbiol 166:375–385. doi:10.1016/j.vetmic.2013.07.001. 23886509 PMC7117237

[45] Bridger JC, Hall GA, Brown JF 1984 Characterization of a calici-like virus (Newbury agent) found in association with astrovirus in bovine diarrhea. Infect Immun 43:133–138. 6418656 10.1128/iai.43.1.133-138.1984 PMC263399

[46] Hall GA, Bridger JC, Brooker BE, Parsons KR, Ormerod E 1984 Lesions of gnotobiotic calves experimentally infected with a calicivirus-like (Newbury) agent. Vet Pathol 21:208–215. doi:10.1177/030098588402100213. 6328722

[47] Smiley JR, Chang KO, Hayes J, Vinje J, Saif LJ 2002 Characterization of an enteropathogenic bovine calicivirus representing a potentially new calicivirus genus. J Virol 76:10089–10098. doi:10.1128/JVI.76.20.10089-10098.2002. 12239283 PMC136553

[48] Jiang X, Wang M, Graham DY, Estes MK 1992 Expression, self-assembly, and antigenicity of the Norwalk virus capsid protein. J Virol 66:6527–6532. 1328679 10.1128/jvi.66.11.6527-6532.1992 PMC240146

[49] Han MG, Wang Q, Smiley JR, Chang KO, Saif LJ 2005 Self-assembly of the recombinant capsid protein of a bovine norovirus (BoNV) into virus-like particles and evaluation of cross-reactivity of BoNV with human noroviruses. J Clin Microbiol 43:778–785. doi:10.1128/JCM.43.2.778-785.2005. 15695679 PMC548067

[50] Woodward MP, Young WW Jr, Bloodgood RA 1985 Detection of monoclonal antibodies specific for carbohydrate epitopes using periodate oxidation. J Immunol Methods 78:143–153. doi:10.1016/0022-1759(85)90337-0. 2580026

[51] Huang P, Farkas T, Marionneau S, Zhong W, Ruvoen-Clouet N, Morrow AL, Altaye M, Pickering LK, Newbur DS, LePendu J, Jiang J 2003 Noroviruses bind to human ABO, Lewis, and secretor histo-blood group antigens: identification of 4 distinct strain-specific patterns. J Infect Dis 188:19–31. doi:10.1086/375742. 12825167

[52] Huang P, Farkas T, Zhong W, Than M, Thornton S, Morrow AL, Jiang X 2005 Norovirus and histo-blood group antigens: demonstration of a wide spectrum of strain specificities and classification of two major binding groups among multiple binding patterns. J Virol 79:6714–6722. doi:10.1128/JVI.79.11.6714-6722.2005. 15890909 PMC1112114

[53] Hafenstein S, Bowman VD, Chipman PR, Kelly CMB, Lin F, Medof ME, Rossmann MG 2007 Interaction of decay-accelerating factor with coxsackievirus B3. J Virol 81:12927–12935. doi:10.1128/JVI.00931-07. 17804498 PMC2169128

[54] Hu L, Crawford SE, Czako R, Cortes-Penfield NW, Smith DF, Le Pendu J, Estes MK, Prasad BV 2012 Cell attachment protein VP8* of a human rotavirus specifically interacts with A-type histo-blood group antigen. Nature 485:256–259. doi:10.1038/nature10996. 22504179 PMC3350622

[55] Huang P, Xia M, Tan M, Zhong W, Wei C, Wang L, Morrow A, Jiang X 2012 Spike protein VP8* of human rotavirus recognizes histo-blood group antigens in a type-specific manner. J Virol 86:4833–4843. doi:10.1128/JVI.05507-11. 22345472 PMC3347384

[56] Liu Y, Huang P, Tan M, Liu M, Liu Y, Biesiada J, Meller J, Castello AA, Jiang B, Jiang X 2012 Rotavirus VP8*: phylogeny, host range, and interaction with histo-blood group antigens. J Virol 86:9899–9910. doi:10.1128/JVI.00979-12. 22761376 PMC3446626

[57] Marionneau S, Cailleau-Thomas A, Rocher J, Moullac-Vaidye BL, Ruvoën N, Clément M, Pendu JL 2001 ABH and Lewis histo-blood group antigens, a model for the meaning of oligosaccharide diversity in the face of a changing world. Biochimie 83:565–573. doi:10.1016/S0300-9084(01)01321-9. 11522384

[58] Zhang XF, Huang Q, Long Y, Jiang X, Zhang T, Tan M, Zhang QL, Huang ZY, Li YH, Ding YQ, Hu GF, Tang S, Dai YC 2015 An outbreak caused by GII.17 norovirus with a wide spectrum of HBGA-associated susceptibility. Sci Rep 5:1–10. doi:10.1038/srep17687. PMC4671059 26639056

[59] Thomas CJ, Surolia A 2000 Mode of molecular recognition of l-fucose by fucose-binding legume lectins. Biochem Biophys Res Commu 268:262–267. doi:10.1006/bbrc.2000.2110. 10679191

[60] Guillon P, Clément M, Sébille V, Rivain JG, Chou CF, Ruvoën-Clouet N, Le Pendu J 2008 Inhibition of the interaction between the SARS-CoV spike protein and its cellular receptor by anti-histo-blood group antibodies. Glycobiology 18:1085–1093. doi:10.1093/glycob/cwn093. 18818423 PMC7108609

[61] Chan MC, Lee N, Hung TN, Kwok K, Cheung K, Tin EK, Lai RW, Nelson EA, Leung TF, Chan PK 2015 Rapid emergence and predominance of a broadly recognizing and fast-evolving norovirus GII.17 variant in late 2014. Nat Commun 6:10061. doi:10.1038/ncomms10061. 26625712 PMC4686777

[62] de Rougemont A, Ruvoen-Clouet N, Simon B, Estienney M, Elie-Caille C, Aho S, Pothier P, Le Pendu J, Boireau W, Belliot G 2011 Qualitative and quantitative analysis of the binding of GII.4 norovirus variants onto human blood group antigens. J Virol 85:4057–4070. doi:10.1128/JVI.02077-10. 21345963 PMC3126233

[63] Trinchera M, Aronica A, Dall'Olio F 2017 Selectin ligands sialyl-Lewis a and sialyl-Lewis x in gastrointestinal cancers. Biology 6:E16. doi:10.3390/biology6010016. 28241499 PMC5372009

[64] Haselhorst T, Fleming FE, Dyason JC, Hartnell RD, Yu X, Holloway G, Santegoest K, Kiefel MJ, Blanchard H, Coulson BS, von Itzstein M 2009 Sialic acid dependence in rotavirus host cell invasion. Nat Chem Biol 5:91–93. doi:10.1038/nchembio.134. 19109595

[65] de Graaf M, Fouchier RA 2014 Role of receptor binding specificity in influenza A virus transmission and pathogenesis. EMBO J 33:823–841. doi:10.1002/embj.201387442. 24668228 PMC4194109

[66] Morizono K, Chen ISY 2011 Receptors and tropisms of envelope viruses. Curr Opin Virol 1:13–18. doi:10.1016/j.coviro.2011.05.001. 21804908 PMC3144558

[67] Schneider-Schaulies J 2000 Cellular receptors for viruses: links to tropism and pathogenesis. J Gen Virol 81:1413–1429. doi:10.1099/0022-1317-81-6-1413. 10811925

[68] Bank-Wolf BR, König M, Thiel HJ 2010 Zoonotic aspects of infections with noroviruses and sapoviruses. Vet Microbiol 140:204–212. doi:10.1016/j.vetmic.2009.08.021. 19773133

[69] Chao DY, Wei JY, Chang WF, Wang J, Wang LC 2012 Detection of multiple genotypes of calicivirus infection in asymptomatic swine in Taiwan. Zoonoses Public Health 59:434–444. doi:10.1111/j.1863-2378.2012.01483.x. 22489630

[70] Di Bartolo Tofani I, Sangenoni G, Ponterio E, Ostanello F, Ruggeri FM 2014 Detection and characterization of porcine caliciviruses in Italy. Arch Virol 159:2479–2484. doi:10.1007/s00705-014-2076-5. 24788843

[71] Keum HO, Moon HJ, Park SJ, Kim HK, Rho SM, Park BK 2009 Porcine noroviruses and sapoviruses on Korean swine farms. Arch Virol 154:1765–1774. J Virol 87:7255–7264. 19812890 10.1007/s00705-009-0501-y

[72] L'Homme Y, Sansregret R, Plante-Fortier E, Lamontagne AM, Lacroix G, Ouardani M, Deschamps J, Simard G, Simard C 2009b Genetic diversity of porcine Norovirus and Sapovirus: Canada, 2005-2007. Arch Virol 154:581–593. 19283338 10.1007/s00705-009-0344-6

[73] Mattison K, Shukla A, Cook A, Pollari F, Friendship R, Kelton D, Bidawid S, Farber JM 2007 Human noroviruses in swine and cattle. Emerg Infect Dis 13:1184–1188. doi:10.3201/eid1308.070005. 17953089 PMC2828081

[74] Reuter G, Biró H, Szucs G 2007 Enteric caliciviruses in domestic pigs in Hungary. Arch Virol 152:611–614. doi:10.1007/s00705-006-0887-8. 17180626

[75] Sugieda M, Nagoka H, Kakishima Y, Ohshita T, Nakamura S, Nakajima S 1998 Detection of Norwalk-like virus genes in the caecum contents of pigs. Arch Virol 143:1215–1221. doi:10.1007/s007050050369. 9687878

[76] Wang QH, Han MG, Cheetham S, Souza M, Funk JA, Saif LJ 2005 Porcine noroviruses related to human noroviruses. Emerg Infect Dis 11:1874–1881. doi:10.3201/eid1112.050485. 16485473 PMC3367634

[77] Cheetham S, Souza M, Meulia T, Grimes S, Han MG, Saif LJ 2006 Pathogenesis of a genogroup II human norovirus in gnotobiotic pigs. J Virol 80:10372–10381. doi:10.1128/JVI.00809-06. 17041218 PMC1641747

[78] Souza M, Azevedo MS, Jung K, Cheetham S, Saif LJ 2008 Pathogenesis and immune responses in gnotobiotic calves after infection with the genogroup II.4-HS66 strain of human norovirus. J Virol 82:1777–1786. doi:10.1128/JVI.01347-07. 18045944 PMC2258707

[79] Farkas T 2015 Rhesus enteric calicivirus surrogate model for human norovirus gastroenteritis. J Gen Virol 96:1504–1514. doi:10.1099/jgv.0.000020. 25502652

[80] Farkas T, Lun CWP 2014 Prevalence of recovirus-neutralizing antibodies in human serum samples. J Clin Microbiol 52:3088–3090. doi:10.1128/JCM.01187-14. 24899037 PMC4136137

[81] Smits SL, Rahman M, Schapendonk CM, van Leeuwen M, Faruque AS, Haagmans BL, Endtz HP, Osterhaus AD 2012 Calicivirus form novel Recovirus genogroup in human diarrhea, Bangladesh. Emerg Infect Dis 18:1192–1195. doi:10.3201/eid1807.120344. 22709854 PMC3376821

[82] Fiege B, Leuthold M, Parra F, Dalton KP, Meloncelli PJ, Lowary TL, Peters T 2017 Epitope mapping of histo blood group antigens bound to norovirus VLPs using STD NMR experiments reveals find details of molecular recognition. Glycoconj J 34:679–689. doi:10.1007/s10719-017-9792-5. 28823097

[83] Nasir W, Frank M, Kunze A, Bally M, Parra F, Nyholm PG, Höök F, Larson G 2017 Histo-blood group antigen presentation is critical for binding of norovirus VLP to glycosphingolipids in model membranes. ACS Chem Biol 12:1288–1296. doi:10.1021/acschembio.7b00152. 28294600

[84] Mesquita JR, Costantini VP, Cannon JL, Lin SC, Nascimento MS, Vinje J 2013 Presence of antibodies against genogroup VI norovirus in humans. Virol J 10:176. doi:10.1186/1743-422X-10-176. 23735311 PMC3680240

[85] Widdowson M, Rockx B, Schepp R, van der Poel WHM, Vinje J, van Duynhoven YT, Koopmans MP 2005 Detection of serum antibodies to bovine norovirus in veterinarians and the general population in the Netherlands. J Med Virol 76:119–128. doi:10.1002/jmv.20333. 15779045

[86] Kim DS, Kang MI, Son KY, Bak GY, Park JG, Hosmillo M, Seo JY, Kim JY, Alfajaro MM, Soliman M, Baek YB, Cho EH, Lee JH, Kwon J, Choi JS, Goodfellow I, Cho KO 2016 Pathogenesis of Korean sapelovirus A in piglets and chicks. J Gen Virol 97:2566–2574. doi:10.1099/jgv.0.000571. 27487773 PMC5078829

[87] Shafren DR, Williams DT, Barry RD 1997 A decay-accelerating factor-binding strain of coxsackievirus B3 requires the coxsackievirus-adenovirus receptor protein to mediate lytic infection of rhabdomyosarcoma cells. J Virol 71:9844–9848. 9371658 10.1128/jvi.71.12.9844-9848.1997 PMC230302

[88] Senger T, Schadlich L, Gissmann L, Muller M 2009 Enhanced papillomavirus-like particle production in insect cells. Virology 388:344–353. doi:10.1016/j.virol.2009.04.004. 19409593

[89] Tan M, Zhong W, Song D, Thornton S, Jiang X 2004 E. coli-expressed recombinant norovirus capsid proteins maintain authentic antigenicity and receptor binding capability. J Med Virol 74:641–649. doi:10.1002/jmv.20228. 15484274

[90] Pelkmans L, Helenius A 2003 Insider information: what viruses tell us about endocytosis. Curr Opin Microbiol 15:414–422. 10.1016/s0955-0674(03)00081-4 12892781

[91] White LJ, Ball JM, Hardy ME, Tanaka TN, Kitamoto N, Estes MK 1996 Attachment and entry of recombinant Norwalk virus capsids to cultured human and animal cell lines. J Virol 70:6589–6597. 8794293 10.1128/jvi.70.10.6589-6597.1996 PMC190699

[92] Azuma Y, Sakanashi M, Matsumono K 2001 The effect of α2,6-linked sialic acid on anti-IgM antibody-induced apoptosis in Ramos cells. Glycoconj J 18:419–424. doi:10.1023/A:1014820316267. 11925509

[93] Seidman D, Hebert KS, Truchan HK, Miller DP, Tegels BK, Marconi RT, Carlyon JA 2015 Essential domains of *Anaplasma phagocytophilum* invasins utilized to infect mammalian host cells. PLoS Pathog 11:e1004669. doi:10.1371/journal.ppat.1004669. 25658707 PMC4450072

[94] Hebert KS, Seidman D, Oki AT, Izac J, Emani S Jr, Oliver LD, Miller DP, Tegels BK, Kannagi R, Marconi RT, Carlyon JA 2017 *Anaplasma marginale* outer membrane protein A is an adhesion that recognizes sialylated and fucosylated glycans and functionally depends on an essential binding domain. Infect Immun 85:e00968-16. doi:10.1128/IAI.00968-16. 27993973 PMC5328490

[95] National Institutes of Health. 1996 Guide for the care and use of laboratory animals. NIH publication no. 85-23. National Institutes of Health, Bethesda, MD.

[96] World Medical Association. 2013 Declaration of Helsinki–ethical principles for medical research involving human subjects. JAMA 310:2191–2194. doi:10.1001/jama.2013.281053. 24141714

